# Potential of measured relative shifts in collision cross section values for biotransformation studies

**DOI:** 10.1007/s00216-023-05063-1

**Published:** 2023-12-02

**Authors:** Christian Lanshoeft, Raphael Schütz, Frédéric Lozac’h, Götz Schlotterbeck, Markus Walles

**Affiliations:** 1 Biomedical Research, PK Sciences, Novartis Pharma AG, Fabrikstrasse 14 (Novartis Campus), 4056 Basel, Switzerland; 2 School of Life Sciences FHNW, Institute for Chemistry and Bioanalytics, University of Applied Sciences and Arts Northwestern Switzerland, Hofackerstrasse 30, 4132 Muttenz, Switzerland; 3 Department of Forensic Chemistry and Toxicology, Institute of Forensic Medicine, University of Basel, Pestalozzistrasse 22, 4056 Basel, Switzerland

**Keywords:** Ion mobility spectrometry mass spectrometry, Collision cross section, Biotransformation assignment, Metabolite mapping, CCSonDemand

## Abstract

Ion mobility spectrometry–mass spectrometry (IMS-MS) separates gas phase ions due to differences in drift time from which reproducible and analyte-specific collision cross section (CCS) values can be derived. Internally conducted in vitro and in vivo metabolism (biotransformation) studies indicated repetitive shifts in measured CCS values (CCS_meas_) between parent drugs and their metabolites. Hence, the purpose of the present article was (i) to investigate if such relative shifts in CCS_meas_ were biotransformation-specific and (ii) to highlight their potential benefits for biotransformation studies. First, mean CCS_meas_ values of 165 compounds were determined (up to *n* = 3) using a travelling wave IMS-MS device with nitrogen as drift gas (^TW^CCS_N2, meas_). Further comparison with their predicted values (^TW^CCS_N2, pred_, Waters CCSonDemand) resulted in a mean absolute error of 5.1%. Second, a reduced data set (*n* = 139) was utilized to create compound pairs (*n* = 86) covering eight common types of phase I and II biotransformations. Constant, discriminative, and almost non-overlapping relative shifts in mean ^TW^CCS_N2, meas_ were obtained for demethylation (− 6.5 ± 2.1 Å^2^), oxygenation (hydroxylation + 3.8 ± 1.4 Å^2^, N-oxidation + 3.4 ± 3.3 Å^2^), acetylation (+ 13.5 ± 1.9 Å^2^), sulfation (+ 17.9 ± 4.4 Å^2^), glucuronidation (N-linked: + 41.7 ± 7.5 Å^2^, O-linked: + 38.1 ± 8.9 Å^2^), and glutathione conjugation (+ 49.2 ± 13.2 Å^2^). Consequently, we propose to consider such relative shifts in ^TW^CCS_N2, meas_ (rather than absolute values) as well for metabolite assignment/confirmation complementing the conventional approach to associate changes in mass-to-charge (*m/z)* values between a parent drug and its metabolite(s). Moreover, the comparison of relative shifts in ^TW^CCS_N2, meas_ significantly simplifies the mapping of metabolites into metabolic pathways as demonstrated.

**Graphical Abstract:**

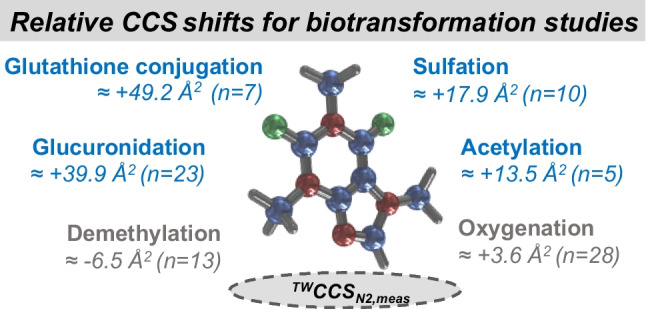

**Supplementary Information:**

The online version contains supplementary material available at 10.1007/s00216-023-05063-1.

## Introduction

Metabolism (biotransformation) refers to the biochemical conversion of endogenous and exogenous compounds from one form to another by specific enzymes aiming to increase their excretion from the body. Biotransformation studies are requested by the US Food and Drug Administration [1, 2], European Medicines Agency [3], or International Conference on Harmonization [4] to bring new drug candidates safely into patients. At discovery stage, metabolic liabilities within a drug candidate need to be investigated to optimize metabolic turnover. Objectives of in vitro and in vivo–conducted biotransformation studies at development stage cover (i) the identification of circulating metabolites in human and selected animal toxicity species, (ii) testing of their pharmacological activity, (iii) assessment of the presence of reactive or toxic metabolites, and (iv) mapping of metabolites into metabolic pathways to elucidate elimination pathways [5–7]. Liquid chromatography–high-resolution mass spectrometry (LC-HRMS) represents the key analytical technology used for biotransformation studies [8–10]. Although LC-HRMS analysis offers a high degree of specificity for metabolite profiling and identification due to obtained retention times, mass-to-charge (*m/z*) ratios of precursor ions, their mass fragmentation, and exact mass measurements, several challenges remain. For instance, isobaric metabolites or structural isomers cannot readily be differentiated from each other solely based on the beforementioned physicochemical parameters due to (i) co-elution, (ii) similar MS, or (iii) MS2 spectral data. Moreover, low abundant in vivo metabolites can hardly be identified when highly abundant co-extracted interferences from matrix components, co-medications, or residual formulation entities are present causing ion suppression and increased detection limits. Lastly, the absence of radiotracers at early drug development stage further impedes a reliable and complete metabolite elucidation. Fortunately, the combination of ion mobility spectrometry (IMS) with mass spectrometry (MS) analysis (IMS-MS) introduces an additional analytical dimension to overcome some of the previously stated hurdles. IMS-MS enables the separation of ions in the gas phase due to differences in size, shape, and charge [11–13]. In the perspective of biotransformation studies, the application of LC-IMS-MS is beneficial in several ways: (i) Elucidation of in vivo metabolites can significantly be facilitated allowing the effective elimination of background ions from matrix components in the MS spectrum [14]. (ii) Positional and structural isomers can readily be separated using either linear [15, 16] or cyclic IMS-MS devices [17–19] exhibiting an increased IMS resolution [20]. (iii) Conformational changes of enzymes upon ligand binding can be assessed [21]. The determination of drift time-derived collision cross section (CCS) values, however, represents the major benefit for metabolism studies [12, 22]. Such robust and analyte-specific parameters allow simple identification and tracking of analytes across investigated matrices, different species, and studies [22]. Such alignment is particularly important when drug development spans over multiple years, compounds have been in-licensed, or biotransformation studies were not entirely conducted in-house (same analytical lab or instrument). Ross et al. [23] previously reported changes in measured CCS values (CCS_meas_) between parent drugs and their in vitro metabolites which created the foundation of our presented work. Their earlier finding agreed with our internal data from various drug development projects. However, we further observed repetitive shifts in relative CCS_meas_ values between parent drugs (or precursors) with their corresponding in vitro or in vivo metabolites. In this publication, we present the outcome of our investigation demonstrating if such relative shifts in CCS_meas_ are biotransformation-specific and highlighting how such relative shifts in CCS_meas_ further benefit biotransformation studies.

## Material and methods

### Chemicals, reagents, and reference material

Formic acid (FA), MS grade water, and acetonitrile (ACN) were obtained from Fisher Chemicals (Loughborough, UK). Adenosine-3′-phosphate 5′ phosphate lithium salt (PAPS), dimethyl sulfoxide, leucine enkephalin acetate salt, reduced L-glutathione (GSH), poly-DL-alanine, and sodium formate were purchased from Sigma-Aldrich (Buchs, Switzerland). Potassium phosphate buffer (0.5 M, pH 7.4) was provided by Thermo Fisher Scientific (Allschwil, Switzerland). Pooled liver S9 fractions were purchased from either BioIVT (West Sussex, UK) or Corning (Woburn, MA, USA). Reference material of investigated compounds was obtained either commercially from various vendors (SI, Table S1) or was synthesized in-house. Due to proprietary reasons, drug names, development codes, batch numbers, or obtained *m/z* values of utilized internal reference material of active development compounds cannot be disclosed. Corresponding material was denoted as either *NVSx* (parent drugs) or *Mx* (metabolites). Moreover, molecular structures of selected internal compounds can only be shown partially.

### Stock and working solutions

Reference material of commercial or internal compounds was first dissolved in dimethyl sulfoxide to obtain stock solutions at 1 mM. Individual stock solutions were further diluted in water/ACN (95/5, v/v) to obtain working solutions at 10 µM containing up to five different compounds. The selection of compounds, which were pooled into individual working solutions, was based on physicochemical properties (molecular weights, polarities, and ionization profiles). Moreover, neither isobaric metabolites (e.g., 10- and 2-hydroxy imipramine) nor metabolites which potentially get back-converted to the parent drug/precursor due to in-source fragmentation (e.g., sulfates and glucuronides) were merged together into the same working solution. No further sample preparation was required for CCS_meas_ determination.

### In vitro incubations

Several parent drugs or precursors were incubated in pooled liver S9 fractions from *CD1 mice*, *Sprague–Dawley rats*, *cynomolgus monkeys*, or *humans* to generate specific metabolites for which no reference material was available (neither commercially nor internally). Deep-frozen pooled liver S9 fractions were first thawed at room temperature and subsequently diluted in potassium phosphate buffer (100 mM, pH 7.4) containing either PAPS (4 mM) or GSH (5 mM) as a cofactor for sulfation or glutathione conjugation, respectively. Following preincubation on a Thermomixer C from Eppendorf (Hamburg, Germany) for 5 min at 37 °C while shaking at 600 rpm, incubations were initiated by adding either the parent drug or precursor (7-hydroxy coumarin, estradiol, raloxifene, and serotonin for sulfation while acalabrutinib, afatinib, branebrutinib, ibrutinib, rociletinib, and spebrutinib were used for GSH conjugation). The total incubation volume was 500 µL with a final protein and substrate concentration of 2 mg/mL and 10 µM, respectively. Following 2 h of incubation at 37 °C and 600 rpm (Thermomixer C), 100 µL of each incubate was drawn and the enzymatic activity was quenched with four volumes of ice-cold ACN. After centrifugation for 15 min at 4 °C and 18,000 g, 200 µL of each supernatant was transferred to a fresh 0.5 mL ProteinLobind tube (Eppendorf). The transferred supernatants were evaporated to dryness under a gentle stream of nitrogen (N_2_) and reconstituted in 50 µL of water/ACN (95/5, v/v). Following brief agitation, each reconstituted supernatant was centrifuged again for 5 min at 4 °C and 18,000 g prior to metabolite profiling.

### Preclinical plasma samples of NVS1

NVS1 was administrated once daily either orally (100 mg/kg for 112 days) or intravenously (5 mg/kg for 111 days) to twenty *Han Wistar* rats or six purebred *beagle* dogs, respectively. In both cases, mixed-gender animals were used. Rats were obtained from Charles River Laboratories (Raleigh, NC, USA) whereas dogs were received from Marshall BioResources (North Rose, NY, USA). Blood samples (300 µL for rat and 1 mL for dog) were drawn at 0.083 (dog only), 0.25 (rat only), 0.5 (dog only) 1, 3, 7, and 24 h post-dose into K2 EDTA-containing tubes. Plasma was harvested following centrifugation for 10 min at 2700 rpm and 4 °C. Regardless of the species, plasma pools (AUC0-24 h) were generated according to the pooling strategy proposed by Hamilton et al. [24]. Metabolite profiling of the protein-precipitated plasma pools was conducted by LC-HRMS analysis (SI, Table S2).

### LC-IMS-MS analysis

CCS_meas_ determination and metabolite profiling were conducted with an Acquity I-Class UPLC system from Waters (Milford, MA, USA) coupled to a Waters Synapt G2-Si HD QTOF high-resolution mass spectrometer. Depending on the sample and experiment, 2–10 µL were injected onto a Waters Acquity UPLC HSS T3 column (2.1 × 150 mm, 1.8 μm) being maintained at 40 °C. The mobile phases consisted of 0.1% FA in water (A) and 0.1% FA in ACN (B). The elution gradient with a flow rate of 0.4 mL/min was set as follows: 0.0–1.0 min, 5% B; 7.8–8.5 min, 95% B; 8.6–10.0 min, 5% B. The minor part of the post-column split (1/5, v/v) was directed towards the mass spectrometer. The capillary voltage of the electrospray ionization (ESI) was either 3 kV (positive mode) or 2 kV (negative mode). The remaining IMS-MS parameters were set as follows: source temperature 120 °C, sampling cone voltage 10 V, cone gas flow rate 20 L/h (N_2_), desolvation temperature 350 °C, desolvation gas flow rate 600 L/h (N_2_), nebulizer gas pressure 6 bar, IMS gas flow rate 90 mL/min (N_2_), IMS wave velocity and height at 500 m/s and 40 V, respectively. HDMS^e^ data were acquired in resolution mode using the continuum data format: full-scan (*m/z* 100–1200) with a scan time of 0.5 s either without (function 1) or with collision energy ramp from 10 to 50 eV in the transfer cell (function 2) of the TriWave device for precursor ion ^TW^CCS_N2_ determination. For product ion ^TW^CCS_N2_ determination, fixed collision energies (either 12 or 25 eV) were applied in the trap cell of the TriWave device on the low instead of high-energy MS trace while maintaining the same IMS-MS parameters as stated above. Mass and ^TW^CCS_N2_ calibrations were fully automated (Waters IntelliStart application in the instrument console) and were conducted with 0.5 mM sodium formate in ACN/water (1/1, v/v) containing 0.1% FA and polyalanine solution at 20.0 µg/mL in ACN/water (1/1, v/v) containing acetaminophen at 5.00 µg/mL, respectively. Utilized reference ^TW^CCS_N2_ values used to convert measured drift times of individual ions into corresponding ^TW^CCS_N2_ values were previously published by Bush et al. [25]. Those values are further provided in the supplementary information (SI, Table S3). The entire LC-IMS-MS system was operated and controlled under Waters Masslynx (v4.2).

### Prediction and determination of measured ^TW^CCS_N2_ values

Mol files of the molecular structure from each investigated compound were generated with ChemDraw (v19.1) from PerkinElmer (Waltham, MA, USA) prior to their upload to CCSonDemand (Waters), a machine learning algorithm for ^TW^CCS_N2_ value prediction (^TW^CCS_N2, pred_) [26, 27]. Measured ^TW^CCS_N2_ values (^TW^CCS_N2, meas_) were determined with Waters Unifi (v1.9.4). For this, accurate mass screening on LC-IMS-MS data was performed with a mass accuracy of 20 ppm. The [M + H]^+^ ion of leucine enkephalin at *m/z* 556.2766 and its drift time were further used for exact mass and CCS correction. Following completion of the post-acquisition data processing, a summary table including identified molecular ions, mass accuracy, retention time, peak intensity, and ^TW^CCS_N2, meas_ was provided for each analyte by Unifi.

### Validation of ^TW^CCS_N2, meas_ values

A reference mix (SI, Table S4) was analyzed whenever a new data set was acquired to validate ^TW^CSS_N2,_ _meas_ values. If several sample sets were acquired in a single run, then three reference mix injections were distributed across the entire sequence to ensure no drift in ^TW^CCS_N2, meas_ over time. If the mean ^TW^CCS_N2, meas_ of each compound in the reference mix was within ± 2% from its previously determined and published value [28], then ^TW^CCS_N2, meas_ determination of the entire sample set was considered acceptable. If not, then ^TW^CCS_N2_ calibration and sample set(s) analysis were repeated.

### Accuracy, precision, and mean absolute error of ^TW^CCS_N2, meas_ versus ^TW^CCS_N2, pred_

The accuracy (% bias) was calculated by dividing the difference between the mean ^TW^CCS_N2, meas_ and ^TW^CCS_N2, pred_ by the ^TW^CCS_N2, pred_ which was further multiplied by a factor of 100. The precision of ^TW^CCS_N2, meas_ was determined by the coefficient of variation (CV): the standard deviation (SD) between individual ^TW^CCS_N2_ measurements divided by the mean ^TW^CCS_N2, meas_ which was multiplied by a factor of 100. The mean absolute error was calculated by dividing the sum of absolute errors (difference between ^TW^CCS_N2, pred_ and mean ^TW^CCS_N2, meas_) by the number of investigated compounds. Error bars displayed in subsequent figures correspond to the obtained SD.

## Results and discussion

In total, 165 compounds were incorporated into our assessment, and their CCS values (^TW^CCS_N2_) were determined following LC-IMS-MS analysis (SI, Table S5). The entire data set, covering an overall mass (*m/z*) and ^TW^CCS_N2_ range of *m/z* 146–862 and 120–280 Å^2^ respectively, was utilized to compare mean measured (^TW^CCS_N2, meas_) with predicted CCS (^TW^CCS_N2, pred_) values (see “Correlation between ^TW^CCS_N2, pred_ and mean ^TW^CCS_N2, meas_ values”). Moreover, a reduced data set (*n* = 139) was further used to verify if the previously observed repetitive shifts in relative ^TW^CCS_N2, meas_ between parent drugs (or precursors) and their metabolites were biotransformation-specific. The selection and inclusion of those compounds for such assessment was conducted based on the possibility of creating compound pairs (*n* = 86; SI, Table S6) covering three common types of phase I biotransformation [hydroxylation (*n* = 19), N-oxidation (*n* = 9), demethylation (*n* = 13)] and five common phase II biotransformations [O-glucuronidation (*n* = 14), N-glucuronidation (*n* = 9), sulfation (*n* = 10), glutathione conjugation (*n* = 7), and acetylation (*n* = 5)]. Although most investigated compounds represented already marketed drugs and their corresponding metabolites, additional internal compounds were included for our assessment to extend the number of compound pairs for certain types of biotransformation. The only prerequisite for internal compound inclusion was to have synthesized reference material with a nuclear magnetic resonance-confirmed structure.

### Reproducibility of ^TW^CCS_N2, meas_ values

Similar to other published data [22, 29–31], the generated analyte-specific ^TW^CCS_N2, meas_ values were highly reproducible over the entire period of our assessment (8 months) which was not only demonstrated with the reference mix used for system suitability testing (SI, Table S4 and Fig. S1) but also with the entire dataset (SI, Table S5). For the latter, the SD between individually determined ^TW^CCS_N2, meas_ values (up to *n* = 3) was maximum 1.8 Å^2^ resulting in a precision ≤ 1.0%.

### Correlation between ^TW^CCS_N2, pred_ and mean ^TW^CCS_N2, meas_ values

**Fig. 1:**
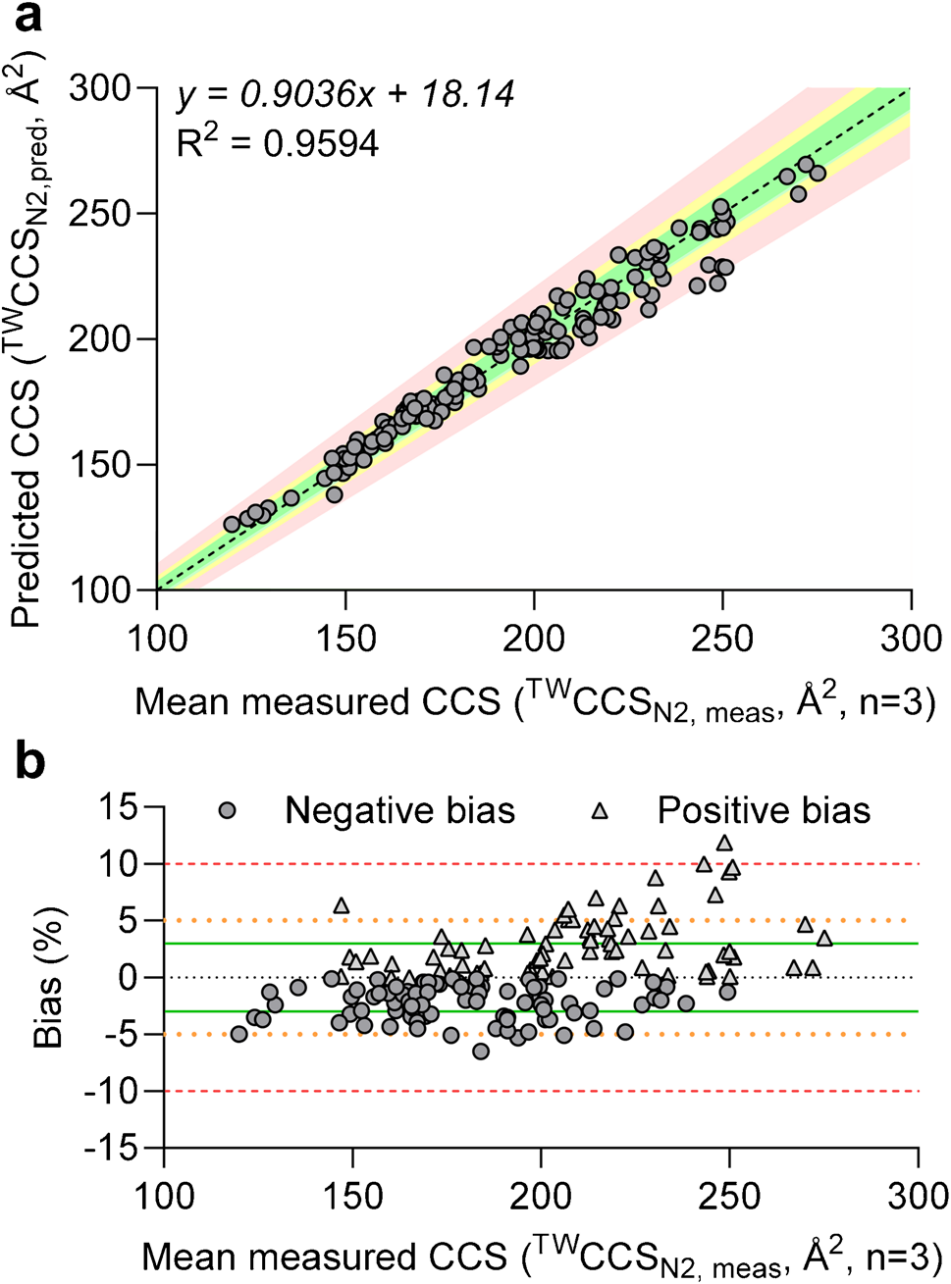
**a** Correlation between predicted (^TW^CCS_N2, pred_) and mean measured CCS values (^TW^CCS_N2, meas_) for 165 investigated compounds. **b** Bias distribution across investigated ^TW^CCS_N2_ range. The colored areas or lines represent the deviation of ± 10% (red/dashed), ± 5% (orange/dotted), and ± 3% (green/solid) from the optimum (dashed black line)

After achieving excellent reproducibility in ^TW^CCS_N2, meas_ values for our entire data set (*n* = 165), a better understanding of its accuracy was required. For this, mean ^TW^CCS_N2, meas_ values were further compared with their ^TW^CCS_N2, pred_ values (Waters CCSonDemand) which overall resulted in a good correlation (*R*^2^ = 0.9594) as depicted in Fig. 1a. The obtained linear regression with a slope of 0.9036 (95% confidence intervals ranging from 0.8748 to 0.9323) and an intercept at + 18.14 (95% confidence intervals ranging from 12.49 to 23.78) was also close to its optimum (dashed black line). On the individual level, the obtained bias between ^TW^CCS_N2, pred_ and mean ^TW^CCS_N2, meas_ ranged from − 6.5 to + 11.9% (SI, Table S5) while the mean absolute error over the entire data set was 5.1%. The bias was within ± 10% (red area/dashed lines in Fig. 1) for 164 of the investigated compounds (99.4%). By further narrowing the acceptance criterion, most of the investigated compounds (87.3%, *n* = 144) met the ± 5% threshold (orange area/dotted lines in Fig. 1) while only 64.8% (*n* = 107) met the most stringent acceptance criterion of ± 3% (green area/solid lines in Fig. 1). It is worth mentioning that additional efforts were conducted to elucidate the origin of obtained bias in our presented data set. Neither the molecular weight (data not shown) nor an increase in ^TW^CCS_N2_ could be identified as a root cause. The latter demonstrated an equal distribution in negative (*n* = 93) and positive bias (*n* = 72) over the investigated ^TW^CCS_N2_ range (Fig. 1b). Hence, additional investigations would be necessary to clarify why certain ^TW^CCS_N2_ values could not be predicted as accurately as other ones. Besides CCSonDemand, other computational tools or algorithms, previously reporting excellent correlations between CCS_pred_ and CCS_meas_ values [32, 33], could further be tested with our publicly shared ^TW^CCS_N2, meas_ values (SI, Table S5 or the separately provided.csv file in the online resources). Such assessment, however, was out of scope for this study. Overall, a high confidence was associated with the generated data set which was used for our assessment of biotransformation-specific relative shifts in ^TW^CCS_N2, meas_ as discussed next.

### Biotransformation-specific relative shifts in mean ^TW^CCS_N2, meas_ values

**Fig. 2:**
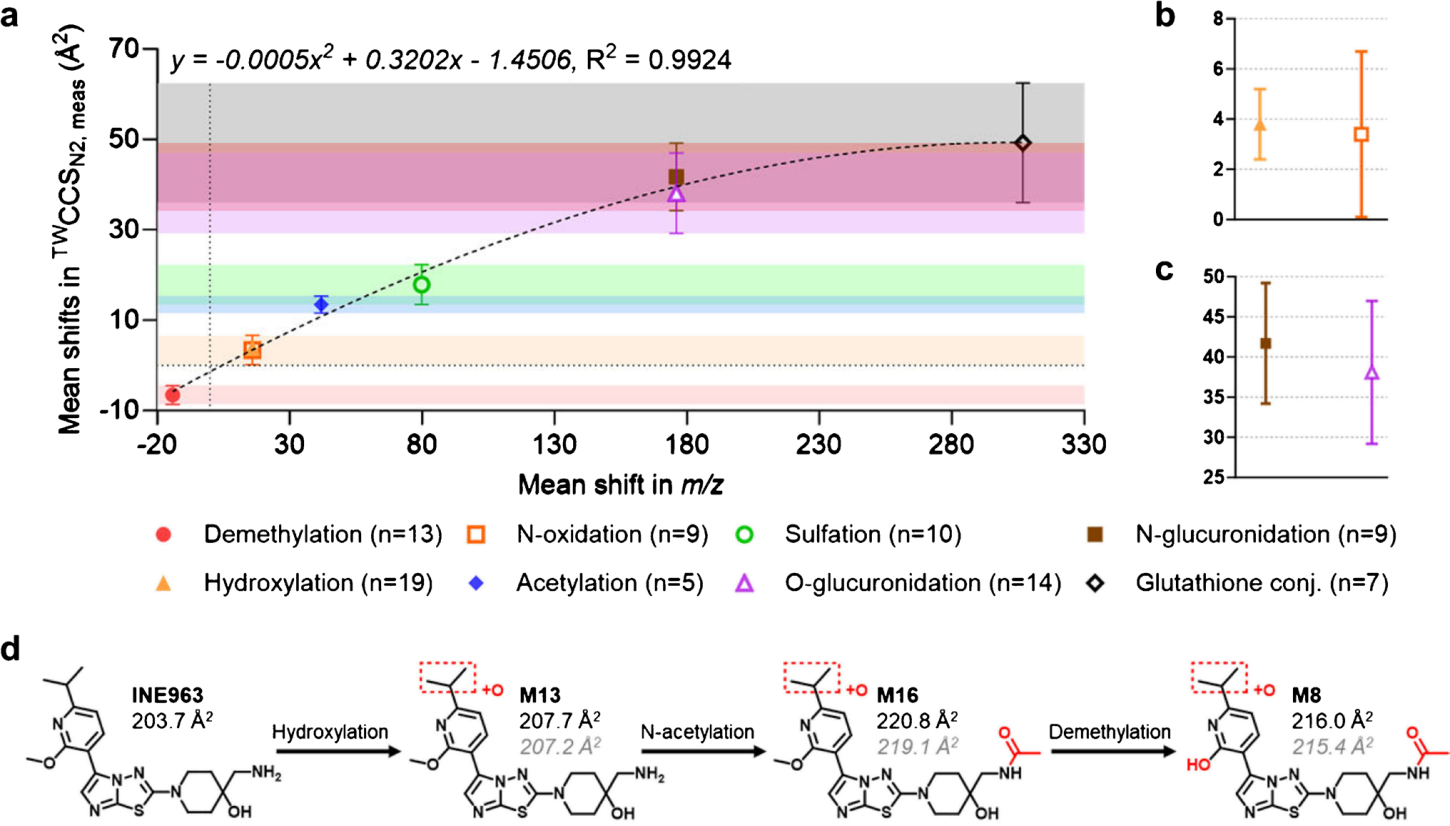
**a** Correlation between mean measured mass changes and relative mean ^TW^CCS_N2, meas_ shifts. Zoom into **b** hydroxylation and N-oxidation as well as **c** O- and N-glucuronidation. **d** Example for ^TW^CCS_N2_ calculation (grey italics values) based on the obtained polynomial regression from **a** in comparison to ^TW^CCS_N2, meas_ values (black values)

An almost linear relationship between shifts in mean ^TW^CCS_N2, meas_ values relative to the increase or decrease in mass was obtained for each type of investigated biotransformation (Fig. 2a). For instance, the elimination of a methyl group from the parent drug (− 14 Da) decreased the mean ^TW^CCS_N2, meas_ relative to the parent drug or precursor by - 6.5 ± 2.1 Å^2^ on average (Table 1). On the other hand, the mean ^TW^CCS_N2, meas_ relative to the parent drug or precursor increased by + 13.5 ± 1.9 Å^2^ on average following acetylation (+ 42 Da) while sulfation (+ 80 Da) increased the mean ^TW^CCS_N2, meas_ in comparison to the parent drug or precursor even further (+ 17.9 ± 4.4 Å^2^). Oxygenation as phase I biotransformation, including hydroxylation (+ 3.8 ± 1.4 Å^2^) and N-oxidation (+ 3.4 ± 3.3 Å^2^), also perfectly fitted into the almost linear relationship (Fig. 2a) which agreed with other published data [23]. However, both phase I biotransformations could not be differentiated from each other purely based on IMS-MS data since similar relative mean shifts in ^TW^CCS_N2, meas_ were observed (Fig. 2b). Fortunately, other experiments such as hydrogen–deuterium exchange [34, 35] or selective reduction of N-oxides to amines with titanium (III) chloride [36] enable the discrimination between both types of biotransformation. Secondary (*n* = 3), tertiary (*n* = 4), and quaternary N-glucuronides (*n* = 2) included in our assessment exhibited on average a slightly higher relative mean shift in ^TW^CCS_N2, meas_ (+ 41.7 ± 7.5 Å^2^) compared to the investigated O-glucuronides (+ 38.1 ± 8.9 Å^2^) with almost identical SDs (Table 1 and Fig. 2c). On some occasions, a much more discriminative relative shift in ^TW^CCS_N2, meas_ values was obtained when the parent drug was either O- or N-glucuronidated (see “Potential to elucidate structural relationships between metabolites”) while the tendency of N-glucuronides exhibiting higher relative shifts in ^TW^CCS_N2, meas_ values compared to O-glucuronides remained constant.

**Table 1:** Obtained mean *m/z* and relative mean ^TW^CCS_N2_ shifts based on measured or predicted CCS values

Biotransformation	Mean measured shift in *m/z*	Relative mean shift in ^TW^CCS_N2_ (Å^2^) based on	
		^TW^CCS_N2, meas_	^TW^CCS_N2, pred_
Demethylation (*n* = 13)	− 14.0159 ± 0.0016	− 6.5 ± 2.1	− 4.8 ± 3.8
Hydroxylation (*n* = 19)	+ 15.9946 ± 0.0013	+ 3.8 ± 1.4	+ 3.3 ± 3.1
N-oxidation (*n* = 9)	+ 15.9948 ± 0.0007	+ 3.4 ± 3.3	+ 4.0 ± 2.2
Acetylation (*n* = 5)	+ 42.0104 ± 0.0008	+ 13.5 ± 1.9	+ 9.9 ± 3.3
Sulfation (*n* = 10)	+ 79.9602 ± 0.0113	+ 17.9 ± 4.4	+ 12.2 ± 4.5
O-glucuronidation (*n* = 14)	+ 176.0313 ± 0.0036	+ 38.1 ± 8.9	+ 35.8 ± 5.8
N-glucuronidation (*n* = 9)	+ 176.0299 ± 0.0061	+ 41.7 ± 7.5	+ 36.4 ± 7.0
Glutathione conjugation (*n* = 7)	+ 306.7958 ± 0.7619	+ 49.2 ± 13.2	+ 52.6 ± 11.3

Corresponding raw data are provided as separate.csv file (see online resource “*Raw data for *Table 1”)

It was further observed that the variation in relative mean shifts in ^TW^CCS_N2, meas_ values was significantly increased for phase II compared to phase I biotransformations which was somehow expected: simple modifications following phase I reactions typically introduce relatively small changes in the molecular structure of the parent drug resulting in fairly consistent shifts in relative ^TW^CCS_N2, meas_ values (except for major dealkylations within the molecule). On the other hand, conjugation of a large and rather flexible residue such as glutathione (tripeptide, + 305 Da) alters the molecular structure significantly which resulted in a mean relative ^TW^CCS_N2, meas_ increase by + 49.2 ± 13.2 Å^2^ on average (Table 1). Two main factors were identified as a potential origin of the increased variation in relative mean ^TW^CCS_N2, meas_ shifts following phase II biotransformation: (i) bulky entities could exist in different gas phase conformations and (ii) the site of conjugation can further influence changes in relative mean ^TW^CCS_N2, meas_ values. For instance, glucuronic acid conjugated to a flexible aliphatic side chain could have a significantly different ^TW^CCS_N2,_ _meas_ compared to its isobaric version where conjugation occurs on a rather rigid aromatic ring system (see example in “Potential to elucidate structural relationships between metabolites”).

Nonetheless, almost non-overlapping bands in relative mean ^TW^CCS_N2, meas_ shifts were obtained for each type of investigated biotransformation (Fig. 2a and Table 1). The plateau towards the upper end of the correlation between the mean measured mass and relative mean ^TW^CCS_N2, meas_ shift was most likely caused by the underestimation of the ^TW^CCS_N2, meas_ values for the glutathione conjugates which rather tend to be multiply charged instead of singly charged. It is noteworthy mentioning here that IMS and the CCS value of a molecule strongly depend on its charge state [29, 37]. For our work, however, the ^TW^CCS_N2, meas_ of a singly charged ion from a metabolite had to be compared with the one of its parent drug (see also “Limitations”). The obtained relative mean ^TW^CCS_N2_ shifts based on ^TW^CCS_N2, meas_ values further agreed with the theoretical relative shifts when taking ^TW^CCS_N2, pred_ values into consideration (Table 1 and SI, Fig. S2).

Based on the obtained polynomial regression (*y* =  *− 0.0005x*^*2*^ + *0.3202x − 1.4506*) between the mean measured mass changes and relative mean shifts in ^TW^CCS_N2, meas_ (Fig. 2a), ^TW^CCS_N2_ values of metabolites could readily be calculated which is illustrated for an internal compound in Fig. 2d: the ^TW^CCS_N2_ of the parent drug (INE963) following hydroxylation was calculated 207.2 Å^2^ (^TW^CCS_N2, calc_) which agreed with the actual ^TW^CCS_N2, meas_ for metabolite M13 (207.7 Å^2^). Even after subsequent N-acetylation and demethylation of M13, resulting in metabolite M8, the ^TW^CCS_N2, calc_ (215.4 Å^2^) was in excellent agreement with the ^TW^CCS_N2, meas_ (216.0 Å^2^). Hence, the discovered biotransformation-specific relative shifts in mean ^TW^CCS_N2, meas_ values (and the combination thereof) can also be considered for metabolite assignment/confirmation (rather than their absolute ^TW^CCS_N2, meas_ values) complementing the conventional approach where changes in *m/z* values are compared. Only for glucuronidation or glutathione conjugation, special care must be taken exhibiting increased variabilities and slightly overlapping bands in relative mean ^TW^CCS_N2, meas_ shifts (Fig. 2a and Table 1).

### Similar biotransformation-specific shifts in relative ^TW^CCS_N2, meas_ obtained on fragment ion level

**Fig. 3:**
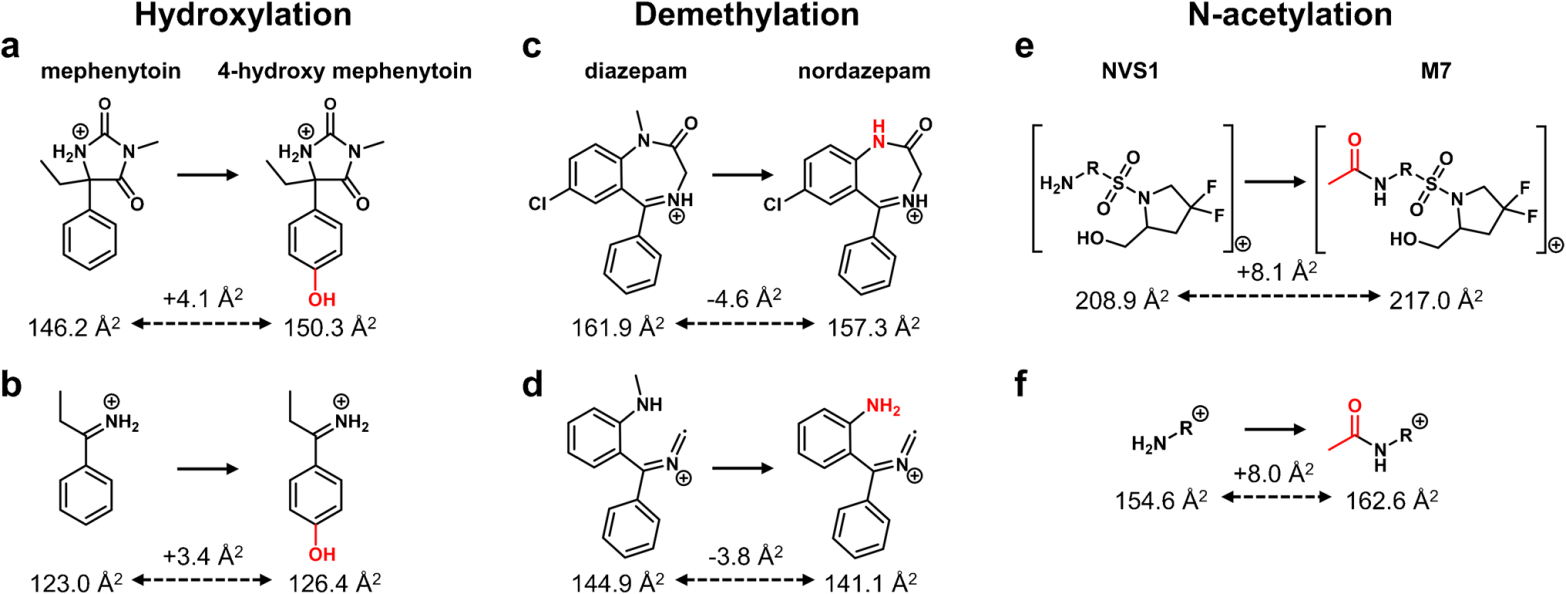
Biotransformation-specific shifts in relative ^TW^CCS_N2, meas_ values obtained either on precursor ion (**a**, **c**, **e**) or fragment ion level (**b**, **d**, **f**) exemplified for hydroxylation, demethylation, and N-acetylation (proposals for charge localization and fragment ion structures are only tentative)

Identical biotransformation-specific shifts in relative ^TW^CCS_N2, meas_ were also obtained on fragment ion level as illustrated for hydroxylation, demethylation, and N-acetylation in Fig. 3. Phase II biotransformations such as sulfation and glucuronidation were not considered for this assessment since the major fragment ion from such metabolites is often only the parent drug or corresponding precursor following the characteristic neutral loss of the sulfate (80 Da, SO_3_) or the glycone (176 Da, C_6_H_8_O_6_), respectively [38–41]. One prerequisite for the assessment of biotransformation-specific shifts in relative ^TW^CCS_N2, meas_ on fragment ion level was the presence of common fragment ions between the metabolite and parent drug or precursor (with and without the biotransformation). The relative shift in ^TW^CCS_N2, meas_ for mephenytoin after hydroxylation, leading to 4-hydroxy mephenytoin, was + 4.1 Å^2^ on precursor ion level (Fig. 3a). An almost identical relative shift (+ 3.4 Å^2^) was obtained after comparing the ^TW^CCS_N2, meas_ values of the major fragment ion for mephenytoin at *m/z* 134 and 4-hydroxy mephenytoin at *m/z* 150 (Fig. 3b). For demethylation, the relative shifts in ^TW^CCS_N2, meas_ between diazepam and nordazepam on precursor and fragment ion level were again in the similar range: − 4.6 Å^2^ on precursor ion level (Fig. 3c) and − 3.8 Å^2^ on fragment ion level (Fig. 3d) considering the fragment ions at *m/z* 222 and *m/z* 208 for diazepam and nordazepam, respectively. The best agreement in biotransformation-specific relative shifts in ^TW^CCS_N2, meas_ determined on precursor (+ 8.1 Å^2^, Fig. 3e) and fragment ion level (+ 8.0 Å^2^, Fig. 3f) was obtained with an internal compound (NVS1) and its N-acetylated metabolite M7. The combination of fragment ion ^TW^CCS_N2, meas_ values, allowing the discrimination of ortho-, para-, or meta-conjugated metabolites due to minor changes in ^TW^CCS_N2, meas_ values [16] and the potential to utilize relative shifts in ^TW^CCS_N2,meas_ values for metabolite mapping purposes (see “Potential to elucidate structural relationships between metabolites”), would represent a powerful analytical tool for future metabolism studies to elucidate structural relationships between positional isomers and their subsequently conjugated metabolites, e.g., glucuronides.

### Potential to elucidate structural relationships between metabolites

**Fig. 4:**
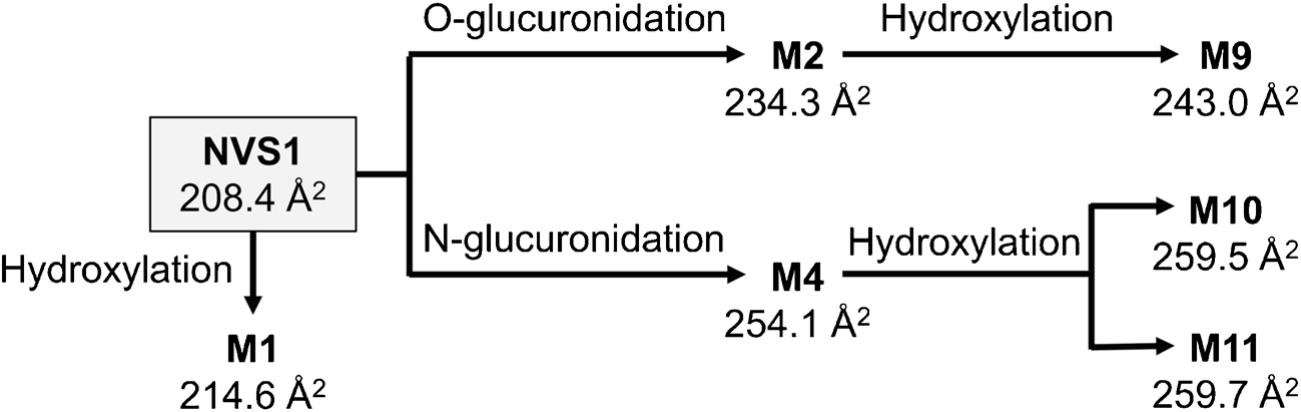
Potential of relative shifts in ^TW^CCS_N2, meas_ values to elucidate structural relationships between metabolites as illustrated with an internal compound (NVS1) and its in vivo obtained hydroxylated and glucuronidated metabolites. Please note that the displayed mapping is based on relative changes in ^TW^CCS_N2, meas_ and not on enzymatic formation (hydroxylation with subsequent glucuronidation)

One major benefit to considering relative shifts in ^TW^CCS_N2, meas_ values for biotransformation studies is the potential to elucidate structural relationships between metabolites in order to design metabolic pathways. During in vivo metabolism assessment of an internal compound (NVS1), several hydroxylated and glucuronidated metabolites were detected in pooled plasma samples (AUC0-24 h) following oral (rat) and intravenous administration (dog), respectively. In both studies, conventional MS fragmentation did not assist in structure elucidation and made the localization of the biotransformation impossible since similar product ion spectra were obtained (data not shown). In contrast, relative shifts in ^TW^CCS_N2, meas_ values provided hints about their structural relationships (Fig. 4). The direct O-glucuronide (M2) was conjugated to a rather flexible side chain whereas the N-glucuronide (M4) was conjugated to a rigid aromatic ring system. Consequently, both direct glucuronides exhibited different ^TW^CCS_N2,_ _meas_ values (234.3 Å^2^ for M2 and 254.1 Å^2^ for M4). By knowing that hydroxylation of NVS1, leading to metabolite M1, increased the ^TW^CCS_N2, meas_ by + 6.2 Å^2^ relative to the parent drug, structural relationships of the detected hydroxylated and glucuronidated metabolites (M9, M10 and M11) relative to both direct glucuronides could readily be elucidated: the differences in ^TW^CCS_N2, meas_ of M10 (+ 25.2 Å^2^) and M11 (+ 25.4 Å^2^) were too high to originate from M2 (O-glucuronide). On the other hand, when the ^TW^CCS_N2, meas_ of M10 (259.5 Å^2^) and M11 (259.7 Å^2^) were compared to the N-glucuronide (M4), then the relative shifts in ^TW^CCS_N2, meas_ values matched with the expected one for NVS1 hydroxylation (+ 5.4 Å^2^ for M10 and + 5.6 Å^2^ for M11). Consequently, M10 and M11 were most likely structurally related to M4. On the other hand, M9 was most likely structurally related to M2.

### Limitations

Despite the above-highlighted benefits to consider relative shifts in ^TW^CCS_N2, meas_ values for biotransformation assignment/confirmation and metabolite mapping purposes, several limitations are also associated with the presented methodology: (i) biotransformation-specific relative shifts in ^TW^CCS_N2, meas_ values could only be obtained when ^TW^CCS_N2, meas_ values of the same ion species were compared with each other. It is not recommended to compare the ^TW^CCS_N2, meas_ of a protonated parent drug with one of its deprotonated metabolites. Moreover, ^TW^CCS_N2, meas_ comparison of a protonated parent drug with the sodium or potassium adduct of its metabolite will neither be successful. (ii) So far only singly charged ions were considered for our assessment. Nevertheless, this already allowed the comparison of a broad range of parent drugs/precursors with their corresponding metabolites since low molecular-weighted drugs with a mass of up to 500–600 Da tend to be mainly singly charged. For future activities, the proposed concept of biotransformation-specific relative shifts in ^TW^CCS_N2, meas_ values may also need to be verified for multiple charged ions. (iii) The current data set is still rather limited and needs to be further extended, e.g., by either adding more compound pairs to already investigated types of biotransformation or by incorporating additional types of biotransformation such as dealkylation reaction causing a significantly greater loss in mass.

## Conclusions

The emerging role of highly reproducible CCS values, as an instrument and analytical method-independent physicochemical property of a molecule, becomes much more evident in various research domains. In our opinion, this should also be considered more frequently for biotransformation studies. Such analyte-specific parameters enable metabolite alignment between various biotransformation studies conducted at diverse analytical labs at different drug development stages. By accessing publicly available computational tools such as Waters CCSonDemand, accurate ^TW^CCS_N2_ value prediction also seems nowadays possible eliminating the need to determine them experimentally.

More importantly, however, is to consider relative shifts in ^TW^CCS_N2, meas_ between a parent drug (or any precursor) and its corresponding metabolite rather than their absolute ^TW^CCS_N2, meas_ values: constant and discriminative relative mean shifts in ^TW^CCS_N2, meas_ values apparently exist as demonstrated for eight different phase I and II biotransformation which were not only obtained on precursor ion but also on fragment ion level. In the perspective of metabolism studies, such biotransformation-specific relative shifts in ^TW^CCS_N2, meas_ would mainly have two major benefits: (i) allowing metabolite identification/confirmation as an orthogonal approach to the conventional comparison of changes in *m/z* values. (ii) The mapping of metabolites in metabolic pathways can significantly be simplified. This would especially be important whenever isobaric metabolites are present, sharing multiple and similar combinations of phase I and II biotransformation but on different molecule locations, which unfortunately cannot be distinguished from each other due to identical MS fragmentation patterns. A simpler metabolite mapping process would be of particular importance for low-abundant circulating metabolites where no synthetic reference material is readily available due to either challenging biosynthesis or resource restrictions. Moreover, in perspective of regulatory guidelines from health authorities, e.g., EMA DDI guidance, demanding to elucidate 90% of detected metabolites in human excreta, the comparison of relative shifts in ^TW^CCS_N2, meas_ to map metabolites correctly would further reduce additional experiments to fully characterize relevant metabolites.

### Supplementary Information

Below is the link to the electronic supplementary material.

Supplementary file1 (DOCX 176 KB)

Supplementary file2 (CSV 14.8 KB)

Supplementary file3 (CSV 9.19 KB)

## Data Availability

All data generated and analyzed during this work are included in this published article and its supplementary information files.
