# Supplementary material for: Potential of measured relative shifts in collision cross section values for biotransformation studies
Source: Anal Bioanal Chem. 2023 Dec 2;416(2):559–68. doi: 10.1007/s00216-023-05063-1 (PMC10761390; doi:10.1007/s00216-023-05063-1)
Supplement: Supplementary file 1 — (DOCX 176 KB) [file 216_2023_5063_MOESM1_ESM.docx]

**Journal of Analytical and Bioanalytical Chemistry (Springer)**

**Supplementary Information (SI)**

**Potential of measured relative shifts in collision cross section values for biotransformation studies**

Christian Lanshoeft^1,*^, Raphael Schütz^2^, Frédéric Lozac’h^1^, Götz Schlotterbeck^2,3^ and Markus Walles^1^

^1^ Novartis Pharma AG, Biomedical Research, PK Sciences, Fabrikstrasse 14 (Novartis Campus), 4056 Basel, Switzerland

^2^ University of Applied Sciences and Arts Northwestern Switzerland, School of Life Sciences FHNW, Institute for Chemistry and Bioanalytics, Hofackerstrasse 30, 4132 Muttenz, Switzerland

^3^ University of Basel, Department of Forensic Chemistry and Toxicology, Institute of Forensic Medicine, Pestalozzistrasse 22, 4056 Basel, Switzerland (current address)

^*^Corresponding author

Novartis Pharma AG, Fabrikstrasse 14 (Novartis Campus), 4056 Basel, Switzerland

Phone: +41 79 647 40 62

Email: christian.lanshoeft[@novartis.com](mailto:olivier.heudi@novartis.com)

# Table of content

Page S-3, Table S1 Detailed information of utilized commercially available reference material

Page S-5, Table S2 LC-HRMS method for *in vivo* metabolite profiling of NVS1

Page S-6, Table S3 Reference *m/z* and ^TW^CCS_N2_ values used for ^TW^CCS_N2_ calibration in ESI+ and ESI-

Page S-6, Table S4 Individual mean ^TW^CCS_N2, meas_ values (n=3) of the reference mix used for system suitability testing

Page S-7, Fig. S1 Plotted mean ^TW^CCS_N2, meas_ values (n=3) of the reference mix over time

Page S-8, Table S5 Summary of predicted (^TW^CCS_N2, pred_), individual and mean measured CCS values (^TW^CCS_N2, meas_) for entire data set (n=165)

Page S-12, Table S6 Summary of investigated compound pairs (n=86) covering eight common types of phase I and II biotransformation

Page S-13, Fig. S2 Comparison of relative mean shifts in ^TW^CCS_N2_ based on predicted and measured values

**Table S1** Detailed information of utilized commercially available reference material

| **Substance** | **CAS No.** | **Vendor** | **Catalogue No.** | **Lot / Batch No.** |
| --- | --- | --- | --- | --- |
| 10-hydroxy imipramine | 796-28-1 | TRC | H939115 | 7-PBS-45-9 |
| 1-hydroxy ibuprofen | 53949-53-4 | Sigma | 32496-10MG | 390960 |
| 1-hydroxy midazolam | 59468-90-5 | Sigma | UC430-5MG | 636-063-3V |
| 2-hydroxy imipramine | 303-70-8 | TRC | H943400 | 3DPP-170-2 |
| 4-acetaminophenyl sulfate potassium salt | 32113-41-0 | TRC | A161230 | 1-SXG-144-1 |
| 4-acetamidophenyl β-D-glucuronide sodium salt | 120595-80-4 | TRC | A158500 | 3-NAV-128-3 |
| 4-hydroxy diclofenac | 64118-84-9 | BD | 451743-5MG | 81083 |
| 4-hydroxy estradiol | 5976-61-4 | Sigma | H4637-1MG | MKCJ3781 |
| 4-hydroxy duloxetine glucuronide sodium salt | 741693-83-4 | TRC | H941805 | 16-QFY-11-1 |
| 4-hydroxy mephenytoin | 61837-65-8 | Sigma | UC126-5MG | BCBD3638V |
| (R)-4-hydroxy propanolol hydrobromide | 10476-53-6 | TRC | H952530 | 4-LXM-131-1 |
| 4-hydroxy propanolol-β-D-glucuronide | 94731-13-2 | TRC | H952535 | 9-SUM-26-1 |
| 4-hydroxy tamoxifen | 68047-06-3 | Novartis | - | NX-4 |
| 5-hydroxy diclofenac | 69002-84-2 | TRC | H825230 | 2-ALN-137-1 |
| 5-hydroxy lansoprazole potassium salt | 1329613-29-7 | TRC | H943711 | 1-RLJ-150-1 |
| 5-hydroxy omeprazole | 92340-57-3 | TRC | H948110 | 7-AKS-23-2 |
| 6-hydroxy chlorzoxazone | 1750-45-4 | Sigma | UC148-5MG | 1385519 |
| 6-β-hydroxy testosterone | 62-99-7 | BD | 451012-5MG | 30979 |
| 7-hydroxy coumarin | 93-35-6 | Chem Ser | MET-104A-1g | 401-52B |
| 7-hydroxy coumarin glucuronide sodium salt | 66695-14-5 | Sigma | UC263-5MG | 1442788 |
| 9'-desmethyl granisetron | 160177-67-3 | TRC | D292040 | 1-APK-24-1 |
| Acalabrutinib | 1420477-60-6 | Novartis | - | NX-2 |
| Acetaminophen | 103-90-2 | TRC | A161220 | 2-GBL-176-1 |
| Acetaminophen glutathione disodium salt | 64889-81-2 | TRC | A161223 | 9-SYQ-6-1 |
| Afatinib | 439081-18-2 | Novartis | - | NX-8 |
| Alprenolol hydrochloride | [13707-88-5](https://www.sigmaaldrich.com/CH/de/search/13707-88-5?focus=products&page=1&perpage=30&sort=relevance&term=13707-88-5&type=cas_number) | Sigma | A0360000 | 1.0 |
| Amitriptyline hydrochloride | 549-18-8 | Sigma | A8404-10G | 086K1138 |
| Amitriptyline N-ß-D-glucuronide | 112806-33-4 | Sigma | A-128-1ML | FN04152102 |
| Amitriptyline N-oxide | 4317-14-0 | TRC | A633355 | 11-MMH-39-1 |
| Atorvastatin calcium salt trihydrate | 344423-98-9 | Sigma | PZ0001-25MG | 020M4701V |
| Branebrutinib | 1912445-55-6 | Novartis | - | NX-1 |
| Bupropion hydrochloride | 31677-93-7 | Sigma | B102-50MG | 019K4710V |
| Chlorzoxazone | 95-25-0 | Sigma | C4397-25G | 070M1256V |
| Chlorzoxazone N-glucuronide | - | Cayman | 19879 | 0489497-1 |
| Clozapine | 5786-21-0 | TRC | C587500 | 5-EOD-183-1 |
| Clozapine N-oxide | 34233-69-7 | TRC | C587520 | 6-NSR-65-1 |
| Coumarin | 91-64-5 | Sigma | C4261-50G | SLBB9006V |
| Dapsone | 80-08-0 | TRC | D193250 | 5-EJB-5-1 |
| Dapsone N-ß-D-glucuronide sodium salt | 54749-81-4 | TRC | D193265 | 7-AKS-158-2 |
| N-desmethyl clozapine | 6104-71-8 | Sigma | D5676-10MG | 153565 |
| Dextromethorphan | 125-71-3 | USP | 1180503 | I3F274 |
| Dextrorphan | 125-73-5 | Sigma | UC205-5MG | 332-121-3 |
| Diazepam | 439-14-5 | TRC | D416855 | 3-YFD-81-1 |
| Diclofenac sodium salt | 15307-79-6 | TRC | D436450 | 1-JLW-79-1 |
| (R)-duloxetine hydrochloride | 136434-34-9 | TRC | D721000 | 23-GHZ-13-1 |
| (Z)-endoxifen | 112093-28-4 | Sigma | SML2368-5MG | 125654 |
| ß-estradiol | 50-28-2 | Sigma | E8875-250MG | SLBB3116V |
| ß-estradiol 3-ß-D-glucuronide sodium salt | 14982-12-8 | Sigma | E2127-10MG | BGBC0580V |
| ß-estradiol-3-sulfate sodium salt | 4999-79-5 | Sigma | E9505-25MG | 104446 |
| Ezetimibe | 163222-33-1 | TRC | E975000 | 10-SCC-14-1 |
| Ezetimibe phenoxy-ß-D-glucuronide | 190448-57-8 | TRC | E975030 | 19-BHW-53-4 |
| Granisetron hydrochloride | 107007-99-8 | TRC | G780000 | 6-XJZ-182-1 |
| Hydroxy bupropion | 106083-71-0 | Sigma | H3167-5MG | 014M4741V |
| Ibrutinib | 936563-96-1 | Novartis | - | NX-3 |

BD: BD Biosciences, Allschwil, Switzerland; Cayman: Cayman Chemicals, Ann Arbor, Michigan, USA; Chem Ser: Chem Service Inc. West Chester, PA, USA; Novartis: Basel, Switzerland; Sigma: Sigma-Aldrich, Buchs, Switzerland; TRC: Toronto Research Chemicals, Toronto, Canada; USP: Rockville, Maryland, USA

**Table S1** continued

| **Substance** | **CAS No.** | **Vendor** | **Catalogue No.** | **Lot / Batch No.** |
| --- | --- | --- | --- | --- |
| Ibuprofen | 15687-27-1 | Novartis | - | CGS003814E |
| Imipramine hydrochloride | 113-52-0 | TRC | I465980 | 23-ABY-121-1 |
| Imipramine N-oxide hydrate | 1215681-42-7 | TRC | I466005 | - |
| Lansoprazole | 103577-45-3 | TRC | L175000 | 15-SSR-133-1 |
| Lansoprazole N-oxide | 213476-12-1 | TRC | L175035 | 3-YMK-103-1 |
| Lansoprazole sulfide | 103577-40-8 | TRC | L175020 | 6-SSR-68-1 |
| Lansoprazole sulfone | 131926-99-3 | TRC | L175026 | 4-AMC-47-4 |
| Lansoprazole sulfone N-oxide | 953787-54-7 | TRC | L175030 | 4-AMC-45-6 |
| Losartan potassium | 124750-99-8 | Fluka | 61188-100MG | BCBH5448V |
| Losartan N-2-glucuronide | 138584-35-7 | TRC | L470495 | 14-BHW-16-1 |
| (S)-mephenytoin | 70989-04-7 | Cayman | 11913 | 0597489-3 |
| Midazolam | 59467-70-8 | TRC | M343000 | 40-GHZ-189-1 |
| N-acetyl sulfamonomethoxine | - | Novartis | - | NX-2 |
| N-acetyl trovafloxacin | - | Novartis | - | NX-1 |
| Naloxone hydrochloride | 465-65-6 | Lipomed | NAL-195-HC-50 | 195.1B0.4 |
| Naloxone-3-β-D-glucuronide hydrate | 22135-79-1 | Lipomed | NAL-450-HY-10 | 450.2B2.1 |
| N-desmethyl tamoxifen hydrochloride | 15917-65-4 | TRC | D293900 | 6-SKS-119-2 |
| Nordazepam | 1088-11-5 | TRC | D291595 | 12-AZC-65-1 |
| Nortestosterone | 434-22-0 | Sigma | N7252 | 129F0649 |
| Omeprazole | 73590-58-6 | Sigma | O104-100MG | BCBN9849V |
| Pantoprazole sodium hydrate | 718635-09-7 | Sigma | P0021-10MG | 113065 |
| Pantoprazole N-oxide | 953787-60-5 | TRC | P183010 | 31-AZC-62-3 |
| Raloxifene hydrochloride | 82640-04-8 | Sigma | R1402-500MG | 036K1054 |
| Raloxifene 4'-glucuronide lithium salt | 182507-22-8 | TLC | R-017 | 1152-049A1 |
| Ranitidine hydrochloride | 66357-59-3 | TRC | R120000 | 11-MIC-57-1 |
| Ranitidine N-oxide | 73857-20-2 | TRC | R120010 | 7-SBK-168-2 |
| Ranitidine S-oxide | 73851-70-4 | TRC | R120015 | 2-MIC-168-1 |
| Ranitidine N,S-dioxide | 1185237-42-6 | TRC | R120020 | 6-SCC-12-1 |
| Repaglinide | 135062-02-1 | TRC | R144500 | 22-SSR-148-1 |
| Repaglinide N-oxide | 121167-81-5 | TRC | R144540 | 2-CHH-64-3 |
| Resveratrol | 501-36-0 | Sigma | R5010-100MG | 044K5206 |
| Resveratrol O-sulfate sodium salt | 858127-11-4 | Cayman | 14942 | 601938-3 |
| Rociletinib | 1374640-70-6 | Novartis | - | NX-1 |
| Salbutamol | 18559-94-9 | Sigma | S8260-25MG | 095K1442 |
| Serotonin | 50-67-9 | Sigma | 14927-25MG | BCBS5678V |
| Spebrutinib | 1202757-89-8 | Novartis | - | NX-1 |
| Sulfamonomethoxine | 1220-83-3 | Novartis | - | NX-1 |
| Tamoxifen citrate salt | 54965-24-1 | Sigma | T9262 | 71K1162 |
| (E,Z)-tamoxifen N-β-D-glucuronide | 794450-92-3 | TRC | T006085 | 4-VKU-12-2 |
| Tamoxifen N-oxide | 75504-34-6 | TRC | T006095 | 35-AZC-40-1 |
| Testosterone | 58-22-0 | Sigma | 46923-250MG-R | SZBD238XV |
| Testosterone β-D-glucuronide | 1180-25-2 | BioCarbosynth | MT08814 | 88141801 |
| Triclosan | 3380-34-5 | Molekula | 73701909-5g | 204168 |
| Triclosan O-sulfate sodium salt | 68508-18-9 | TRC | T774265 | 6-VKU-165-2 |
| Triclosan O-β-D-glucuronide sodium salt | 63156-12-7 | TRC | T774260 | 3-RTU-167-1 |
| Trovafloxacin | 147059-72-1 | Novartis | - | NX-1 |
| Verapamil hydrochloride | 152-11-4 | Sigma | V4629-5G | 058K1148 |

BioCarbosynth: Biosynth Carbosynth, Staad, Switzerland; Cayman: Cayman Chemicals, Ann Arbor, Michigan, USA; Fluka: Buchs, Switzerland; Lipomed: Lipomed GmbH, Weil am Rhein, Deutschland; Molekula: Molekula GmbH: München, Germany; Novartis: Basel, Switzerland; Sigma: Sigma-Aldrich, Buchs, Switzerland; TLC standards: Ontario, Canada; TRC: Toronto Research Chemicals, Toronto, Canada

**Table S2** LC-HRMS method for *in vivo* metabolite profiling of NVS1

| **Liquid chromatography** | | | |
| --- | --- | --- | --- |
| *Instrumentation* | Waters Acquity I-Class UPLC | | |
| *Column* | Waters Acquity UPLC BEH Phenyl, 2.1 x 150 mm, 1.7 µm | | |
| *Column temperature* | 80 ˚C | | |
| *Injection volume* | 20 µL (partial loop) | | |
| *Mobile phases* | A: 10 mM ammonium carbamate in water (pH 9.0)  B: Acetonitrile | | |
| *Flow rate* | 0.4 mL/min | | |
| *Gradient* | Time (min) | | %B |
|  | 0.0 - 1.0: | | 5 |
|  | 22.0: | | 30 |
|  | 28.0 - 35.0: | | 95 |
|  | 35.5 - 40.0: | | 5 |
| **Mass spectrometric detection** | | | |
| *Instrumentation* | Waters Synapt G2-Si HD QTOF high-resolution mass spectrometer | | |
| *Ionization* | Electrospray in positive mode | | |
| *Ion source* | Capillary voltage: | 3.0 kV | |
|  | Source temperature: | 120 ˚C | |
|  | Sampling cone voltage: | 20.0 V | |
|  | Cone gas flow rate: | 20.0 L/h (nitrogen) | |
|  | Desolvation temperature: | 350 ˚C | |
|  | Desolvation gas flow rate: | 600 L/h (nitrogen) | |
|  | Nebuliser gas pressure: | 6 bar | |
| *Analyzer / data format / scan time* | Resolution mode / continuum / 0.4 s | | |
| *Scanning mode* | HDMS^e^ acquisition mode: full-scan (*m/z* 50-1200) without (function 1) and with collision energy ramp from 10 to 50 eV in the transfer cell (function 2). Function 3 was utilized for lock spray data acquisition (leucine enkephalin at 20.0 ng/mL in acetonitrile/water (1/1, v/v) + 0.1% formic acid). The [M+H]^+^ of leucine enkephalin at *m/z* 556.2766 was used for exact mass correction in Waters Unifi (v1.9.4). | | |
| **Ion mobility** |  | | |
| *Drift gas flow rate* | 90 mL/min (nitrogen) | | |
| *CCS calibration* | Conducted with polyalanine solution at 20.0 µg/mL in acetonitrile/water (1/1, v/v) containing also acetaminophen at 5.00 µg/mL. | | |

**Table S3** Reference *m/z* and ^TW^CCS_N2_ values used for ^TW^CCS_N2_ calibration in ESI+ and ESI-

| **ESI+** (13 ions used for calibration) | | **ESI-** (14 ions used for calibration) | | |
| --- | --- | --- | --- | --- |
| *m/z* | ^TW^CCS_N2_ | | *m/z* | ^TW^CCS_N2_ |
| 152.1 | 130.4 | | 230.1 | 150.0 |
| 232.1 | 151.0 | | 301.2 | 165.0 |
| 303.2 | 166.0 | | 372.2 | 179.0 |
| 374.2 | 181.0 | | 443.2 | 195.0 |
| 445.2 | 195.0 | | 514.3 | 209.0 |
| 516.3 | 211.0 | | 585.3 | 223.0 |
| 587.3 | 228.0 | | 656.3 | 238.0 |
| 658.4 | 243.0 | | 727.4 | 253.0 |
| 729.4 | 256.0 | | 798.4 | 267.0 |
| 800.4 | 271.0 | | 869.5 | 279.0 |
| 871.5 | 282.0 | | 940.5 | 294.0 |
| 942.5 | 294.0 | | 1011.5 | 308.0 |
| 1013.5 | 306.0 | | 1082.6 | 322.0 |
| - | - | | 1153.6 | 335.0 |

**Table S4** Individual mean ^TW^CCS_N2, meas_ values (n=3) of the reference mix used for system suitability testing

| **Date** | **4-hydroxy diclofenac** | **clozapine**  **N-oxide** | **acetaminophen** | **4-acetamino-phenyl sulfate** | **acetaminophen glutathione** | **4-acetamidophenyl β‑D-glucuronide** |
| --- | --- | --- | --- | --- | --- | --- |
| 01.04.22 | 160.3^a^ | 178.6^a^ | 126.6^a^ | 147.6^a^ | 199.3^a^ | 169.0^a^ |
| 04.04.22 | 160.6^b^ | 179.3^b^ | 126.8^b^ | 147.8^b^ | 200.6^b^ | 169.1^a^ |
| 07.04.22 | 161.3 | 179.9 | 127.1 | 147.9 | 201.3 | 169.3 |
| 08.04.22 | 161.5^b^ | 180.0^b^ | 127.1^a^ | 148.0^b^ | 202.7^b^ | 169.4^b^ |
| 21.04.22 | 161.4 | 180.0 | 127.0^b^ | 147.9 | 202.7 | 169.4 |
| 22.04.22 | 161.3 | 179.8 | 127.0 | 147.9 | 199.2 | 169.3 |
| 26.04.22 | 161.4 | 179.8 | 126.9 | 147.9 | 201.5 | 169.3 |
| 29.04.22 | 161.6 | 180.0 | 127.0 | 148.0 | 197.9 | 169.4 |
| 05.05.22 | 160.8 | 179.6 | 126.5 | 147.5 | 200.3 | 168.9 |
| 09.05.22 | 160.8 | 179.7 | 126.8 | 147.8 | 201.3 | 169.3 |
| 16.05.22 | 161.1 | 179.7 | 126.7 | 147.7 | 201.3 | 169.3 |
| 17.05.22 | 160.8 | 179.7 | 126.8 | 147.8 | 200.7 | 169.3 |
| 01.06.22 | 161.2 | 179.8 | 127.0 | 147.8 | 201.5 | 168.8 |
| 10.06.22 | 161.0 | 179.4 | 127.0 | 147.7 | 199.2 | 169.2 |
| 13.06.22 | 160.8 | 179.4 | 126.5 | 147.4 | 198.7 | 168.9 |
| 27.06.22 | 161.2 | 179.7 | 126.8 | 147.7 | 201.7 | 169.3 |
| 16.12.22 | 159.9^a^ | 178.6^a^ | 126.4^a^ | 147.4^a^ | 198.3^a^ | 168.9^a^ |
| **Mean±95% CI^c^** | **161.0±0.2** | **179.6±0.2** | **126.8±0.1** | **147.8±0.1** | **200.5±0.8** | **169.2±0.1** |
| SD | 0.4 | 0.4 | 0.2 | 0.2 | 1.5 | 0.2 |
| CV | 0.3 | 0.2 | 0.2 | 0.1 | 0.7 | 0.1 |
| ^a^ n=1, ^b^ n=2, ^c^ the 95% confidence interval (CI) was calculated according to $t \frac{SD}{\sqrt{n}}$ where t is the t value with the degrees of freedom (n-1) for the sample size n | | | | | | |

**Fig. S1** Plotted mean ^TW^CCS_N2, meas_ values (n=3) of the reference mix over time

**
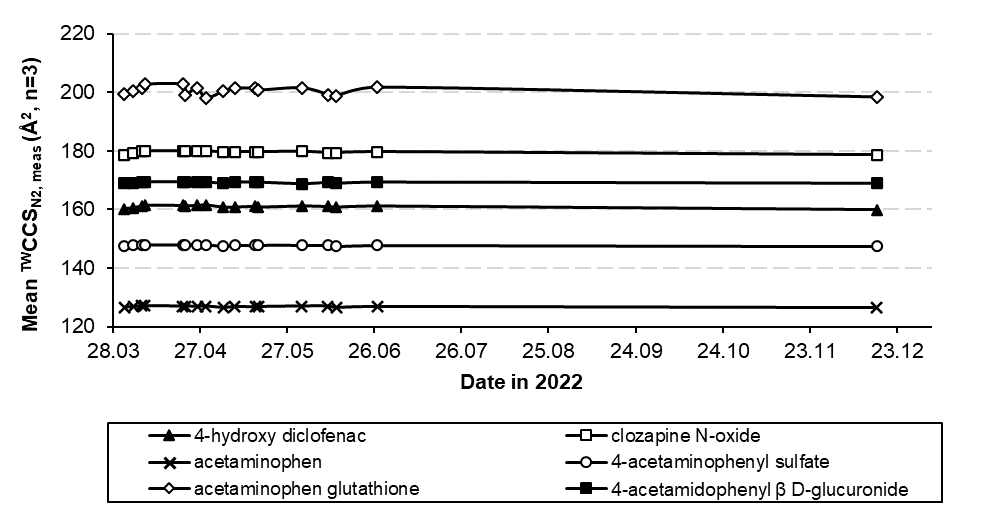
**

**Table S5** Summary of predicted (^TW^CCS_N2, pred_), individual and mean measured CCS values (^TW^CCS_N2, meas_) for the entire data set (n=165)

| **Compound** | **Biotransformation** | **Ion** | ***m/z*** | **^TW^CCS_N2, pred_**  **(Å^2^)^a^** | **^TW^CCS_N2, meas_ (Å^2^)** | | | | | | **Bias**  **(%)^d^** |
| --- | --- | --- | --- | --- | --- | --- | --- | --- | --- | --- | --- |
|  |  |  |  |  | 1 | 2 | 3 | **Mean** | SD^b^ | CV^c^ |  |
| 10-hydroxy imipramine | Hydroxylation | [M+H]^+^ | 297.1974 | **172.7** | 166.5 | 166.5 | 166.3 | **166.4** | 0.1 | 0.1 | -3.6 |
| 1-hydroxy ibuprofen | Hydroxylation | [M-H]^-^ | 221.1188 | **154.4** | 149.5 | 149.5 | 149.3 | **149.4** | 0.1 | 0.1 | -3.2 |
| 1-hydroxy midazolam | Hydroxylation | [M+H]^+^ | 342.0813 | **175.3** | 169.5 | 169.5 | 169.2 | **169.4** | 0.2 | 0.1 | -3.4 |
| 2-hydroxy imipramine | Hydroxylation | [M+H]^+^ | 297.1977 | **171.9** | 169.5 | 169.6 | 169.3 | **169.4** | 0.2 | 0.1 | -1.4 |
| 4-acetaminophenyl sulfate | Sulfation | [M+H]^+^ | 232.0272 | **146.6** | 148.9 | 149.5 | 149.4 | **149.3** | 0.3 | 0.2 | 1.8 |
| 4-acetamidophenyl β-D-glucuronide | O-glucuronidation | [M+H]^+^ | 328.1027 | **171.1** | 169.3 | 170.4 | 170.2 | **170.0** | 0.6 | 0.3 | -0.7 |
| 4-hydroxy diclofenac | Hydroxylation | [M+H]^+^ | 312.0187 | **166.3** | 161.8 | 161.2 | 161.6 | **161.5** | 0.3 | 0.2 | -2.9 |
| 4-hydroxy estradiol | Hydroxylation | [M-H]^-^ | 287.1659 | **171.3** | 165.6 | 165.4 | 165.5 | **165.5** | 0.1 | 0.1 | -3.4 |
| 4-hydroxy glucuronide duloxetine | Hydroxylation + glucuronidation | [M+H]^+^ | 490.1529 | **212.6** | 207.8 | 207.9 | 207.2 | **207.6** | 0.4 | 0.2 | -2.3 |
| 4-hydroxy mephenytoin | Hydroxylation | [M+H]^+^ | 235.1086 | **148.9** | 151.0 | 150.9 | 150.9 | **150.9** | 0.1 | <0.1 | 1.4 |
| 4-hydroxy propranolol | Hydroxylation | [M+H]^+^ | 276.1601 | **167.2** | 159.9 | 160.2 | 159.7 | **159.9** | 0.3 | 0.2 | -4.3 |
| 4-hydroxy propranolol glucuronide | Hydroxylation + O-glucuronidation | [M+H]^+^ | 452.1923 | **199.9** | 196.3 | 199.1 | 199.1 | **198.2** | 1.6 | 0.8 | -0.9 |
| 4-hydroxy tamoxifen | Hydroxylation | [M+H]^+^ | 388.2273 | **203.1** | 199.4 | 199.9 | 199.9 | **199.7** | 0.3 | 0.1 | -1.7 |
| 5-hydroxy diclofenac | Hydroxylation | [M+H]^+^ | 312.0177 | **164.9** | 161.2 | 161.5 | 161.0 | **161.2** | 0.3 | 0.2 | -2.2 |
| 5-hydroxy lansoprazole | Hydroxylation | [M+H]^+^ | 386.0783 | **185.8** | 184.3 | 184.3 | 184.4 | **184.3** | 0.1 | <0.1 | -0.8 |
| 5-hydroxy omeprazole | Hydroxylation | [M+H]^+^ | 362.1181 | **184.4** | 183.0 | 183.1 | 182.8 | **183.0** | 0.1 | 0.1 | -0.8 |
| 6-hydroxy chlorzoxazone | Hydroxylation | [M+H]^+^ | 185.9965 | **132.7** | 129.5 | 129.4 | 129.5 | **129.5** | 0.1 | <0.1 | -2.4 |
| 6-β-hydroxy testosterone | Hydroxylation | [M+H]^+^ | 305.2120 | **174.2** | 173.2 | 172.9 | 173.3 | **173.1** | 0.2 | 0.1 | -0.6 |
| 7-hydroxy coumarin | Hydroxylation | [M+H]^+^ | 163.0402 | **128.6** | 124.1 | 124.1 | 124.2 | **124.1** | 0.1 | <0.1 | -3.5 |
| 7-hydroxy coumarin glucuronide | Hydroxylation + O-glucuronidation | [M+H]^+^ | 339.0719 | **172.2** | 173.3 | 173.3 | 173.3 | **173.3** | <0.1 | <0.1 | 0.6 |
| 7-hydroxy coumarin sulfate^e^ | Hydroxylation + sulfation | [M+H]^+^ | 242.9966 | **144.6** | 144.8 | 143.9 | 144.7 | **144.5** | 0.5 | 0.3 | -0.1 |
| 9'-desmethyl granisetron | Demethylation | [M+H]^+^ | 299.1869 | **174.7** | 179.0 | 178.6 | 179.1 | **178.9** | 0.3 | 0.1 | 2.4 |
| Acalabrutinib | Parent drug | [M+H]^+^ | 466.1984 | **211.8** | 230.6 | 230.3 | 230.5 | **230.4** | 0.1 | 0.1 | 8.8 |
| Acalabrutinib glutathione^e^ | Glutathione conjugation | [M+H]^+^ | 773.2829 | **264.7** | 267.3 | 266.8 | 267.1 | **267.0** | 0.2 | 0.1 | 0.9 |
| Acetaminophen | Parent drug | [M+H]^+^ | 152.0705 | **129.7** | 126.9 | 128.9 | 128.3 | **128.0** | 1.0 | 0.8 | -1.3 |
| Acetaminophen glutathione | Glutathione conjugation | [M+H]^+^ | 457.1385 | **199.4** | 200.2 | 200.8 | 200.1 | **200.4** | 0.4 | 0.2 | 0.5 |
| Afatinib | Parent drug | [M+H]^+^ | 486.1696 | **217.4** | 231.2 | 231.0 | 231.3 | **231.2** | 0.2 | 0.1 | 6.3 |
| Afatinib glutathione^e^ | Glutathione conjugation | [M+H]^+^ | 793.2530 | **257.8** | 270.2 | 269.9 | 269.9 | **270.0** | 0.2 | 0.1 | 4.7 |
| Alprenolol | Parent drug | [M+H]^+^ | 250.1818 | **160.3** | 157.9 | 158.6 | 158.0 | **158.2** | 0.4 | 0.3 | -1.3 |
| Amitriptyline | Parent drug | [M+H]^+^ | 278.1914 | **166.6** | 164.5 | 164.9 | 164.3 | **164.6** | 0.3 | 0.2 | -1.2 |
| Amitriptyline N-ß-D-glucuronide | N-glucuronidation | [M+H]^+^ | 454.2224 | **208.8** | 201.9 | 200.8 | 200.8 | **201.2** | 0.6 | 0.3 | -3.7 |
| Amitriptyline N-oxide | N-oxidation | [M+H]^+^ | 294.1861 | **172.0** | 170.3 | 170.5 | 170.5 | **170.4** | 0.1 | 0.1 | -0.9 |
| Atorvastatin | Parent drug | [M+H]^+^ | 559.2605 | **233.2** | 233.7 | 233.9 | 233.5 | **233.7** | 0.2 | 0.1 | 0.2 |
| Branebrutinib | Parent drug | [M+H]^+^ | 371.1876 | **193.6** | 191.1 | 191.0 | 191.5 | **191.2** | 0.3 | 0.1 | -1.2 |
| Branebrutinib glutathione^e^ | Glutathione conjugation | [M+H]^+^ | 678.2714 | **244.2** | 238.6 | 238.4 | 238.5 | **238.5** | 0.1 | <0.1 | -2.3 |
| Bupropion | Parent drug | [M+H]^+^ | 240.1160 | **158.4** | 155.7 | 155.7 | 155.7 | **155.7** | <0.1 | <0.1 | -1.7 |
| Chlorzoxazone | Parent drug | [M+H]^+^ | 170.0016 | **131.0** | 126.2 | 126.2 | 126.1 | **126.2** | 0.1 | <0.1 | -3.7 |
| Chlorzoxazone N-glucuronide | N-glucuronidation | [M+H]^+^ | 346.0153 | **175.4** | 175.8 | 175.8 | 175.7 | **175.8** | 0.1 | <0.1 | 0.2 |
| Clozapine | Parent drug | [M+H]^+^ | 327.1375 | **178.4** | 177.6 | 178.1 | 177.5 | **177.8** | 0.3 | 0.2 | -0.4 |
| Clozapine N-oxide | N-oxidation | [M+H]^+^ | 343.1319 | **181.7** | 179.7 | 180.3 | 180.0 | **180.0** | 0.3 | 0.2 | -0.9 |
| Coumarin | Parent drug | [M+H]^+^ | 147.0456 | **126.3** | 119.9 | 119.9 | 120.0 | **119.9** | 0.1 | <0.1 | -5.0 |
| ^a^ Waters CCSonDemand, ^b^ SD: standard deviation, ^c^ CV: coefficient of variation, ^d^ Bias (%) = 100 x (mean ^TW^CCS_N2, meas_ - ^TW^CCS_N2, pred_) / ^TW^CCS_N2, pred_, ^e^ generated following *in vitro* incubation of parent drug / precursor | | | | | | | | | | | |

**Table S5** continued

| **Compound** | **Biotransformation** | **Ion** | ***m/z*** | **^TW^CCS_N2, pred_**  **(Å^2^)^a^** | **^TW^CCS_N2, meas_ (Å^2^)** | | | | | | **Bias**  **(%)^d^** |
| --- | --- | --- | --- | --- | --- | --- | --- | --- | --- | --- | --- |
|  |  |  |  |  | 1 | 2 | 3 | Mean | SD^b^ | CV^c^ |  |
| Dapsone | Parent drug | [M+H]^+^ | 249.0702 | **152.4** | 150.0 | 149.8 | 149.4 | **149.7** | 0.3 | 0.2 | -1.7 |
| Dapsone N-glucuronide | N-glucuronidation | [M+H]^+^ | 425.1018 | **196.5** | 207.3 | 207.7 | 207.3 | **207.4** | 0.2 | 0.1 | 5.6 |
| Desmethyl clozapine | Demethylation | [M+H]^+^ | 313.1219 | **173.0** | 172.2 | 172.1 | 172.3 | **172.2** | 0.1 | 0.1 | -0.5 |
| Dextromethorphan | Parent drug | [M+H]^+^ | 272.2018 | **165.9** | 164.0 | 163.3 | 162.9 | **163.4** | 0.6 | 0.3 | -1.5 |
| Dextrorphan | Demethylation | [M+H]^+^ | 258.1862 | **161.8** | 159.5 | 159.5 | 159.5 | **159.5** | <0.1 | <0.1 | -1.4 |
| Diazepam | Parent drug | [M+H]^+^ | 285.0798 | **162.9** | 161.6 | 161.5 | 161.7 | **161.6** | 0.1 | 0.1 | -0.8 |
| Diclofenac | Parent drug | [M+H]^+^ | 296.0240 | **157.0** | 156.9 | 157.2 | 156.1 | **156.7** | 0.6 | 0.4 | -0.2 |
| Duloxetine | Parent drug | [M+H]^+^ | 298.1266 | **169.4** | 168.7 | 168.7 | 168.8 | **168.7** | 0.1 | <0.1 | -0.4 |
| EMA1036 | Demethylation | [M-H]^-^ | 492.1820 | **220.5** | 220.4 | 220.1 | 220.6 | **220.4** | 0.3 | 0.1 | -0.1 |
| EMA1322 | O-debenzylation + demethylation | [M-H]^-^ | 402.1351 | **196.9** | 190.4 | 190.1 | 190.2 | **190.2** | 0.2 | 0.1 | -3.4 |
| EMA1323 | O-debenzylation | [M-H]^-^ | 416.1500 | **197.2** | 196.8 | 196.8 | 196.8 | **196.8** | <0.1 | <0.1 | -0.2 |
| Endoxifen | Demethylation | [M+H]^+^ | 374.2119 | **198.2** | 192.3 | 192.3 | 189.1 | **191.2** | 1.8 | 1.0 | -3.5 |
| Estradiol | Parent drug | [M-H]^-^ | 271.1710 | **169.5** | 165.6 | 165.6 | 165.5 | **165.6** | 0.1 | <0.1 | -2.3 |
| Estradiol-3-glucuronide | O-glucuronidation | [M-H]^-^ | 447.2028 | **208.2** | 213.5 | 212.1 | 213.3 | **213.0** | 0.8 | 0.4 | 2.3 |
| Estradiol-3-sulfate | Sulfation | [M-H]^-^ | 351.1271 | **180.2** | 184.8 | 186.4 | 184.8 | **185.3** | 0.9 | 0.5 | 2.8 |
| Ezetimibe | Parent drug | [M-H]^-^ | 408.1422 | **201.2** | 199.5 | 200.2 | 199.2 | **199.6** | 0.5 | 0.3 | -0.8 |
| Ezetimibe phenoxy-β-glucuronide | O-glucuronidation | [M-H]^-^ | 584.1735 | **234.1** | 231.8 | 231.9 | 231.9 | **231.9** | 0.1 | <0.1 | -1.0 |
| Granisetron | Parent drug | [M+H]^+^ | 313.2029 | **183.4** | 184.2 | 184.4 | 183.7 | **184.1** | 0.4 | 0.2 | 0.4 |
| Hydroxy bupropion | Hydroxylation | [M+H]^+^ | 256.1106 | **158.6** | 160.5 | 160.4 | 160.5 | **160.5** | 0.1 | <0.1 | 1.2 |
| Ibrutinib | Parent drug | [M+H]^+^ | 441.2030 | **205.8** | 212.9 | 213.6 | 212.7 | **213.1** | 0.5 | 0.2 | 3.5 |
| Ibrutinib glutathione^e^ | Glutathione conjugation | [M+H]^+^ | 748.2866 | **266.0** | 275.4 | 275.1 | 275.1 | **275.2** | 0.2 | 0.1 | 3.5 |
| Ibuprofen | Parent drug | [M-H]^-^ | 205.1227 | **152.6** | 146.5 | 146.5 | 146.4 | **146.5** | 0.1 | <0.1 | -4.0 |
| Icenticaftor | Parent drug | [M-H]^-^ | 360.0792 | **170.8** | 165.8 | 166.4 | 166.5 | **166.2** | 0.4 | 0.2 | -2.7 |
| Icenticaftor M1 | N-glucuronidation | [M-H]^-^ | 536.1107 | **210.1** | 204.1 | 201.3 | 201.4 | **202.3** | 1.6 | 0.8 | -3.7 |
| Imipramine | Parent drug | [M+H]^+^ | 281.2021 | **165.2** | 165.2 | 165.4 | 164.8 | **165.1** | 0.3 | 0.2 | <0.1 |
| Imipramine N-oxide | N-oxidation | [M+H]^+^ | 297.1972 | **169.6** | 169.0 | 168.8 | 169.1 | **169.0** | 0.1 | 0.1 | -0.4 |
| Infigratinib M1 (CQM157) | Dealkylation | [M+H]^+^ | 463.1047 | **206.4** | 213.2 | 213.3 | 213.1 | **213.2** | 0.1 | 0.1 | 3.3 |
| Infigratinib M35 | Dealkylation + acetylation | [M+H]^+^ | 505.1147 | **219.7** | 228.4 | 229.0 | 228.4 | **228.6** | 0.3 | 0.1 | 4.1 |
| INE963 | Parent drug | [M+H]^+^ | 403.1912 | **195.4** | 203.3 | 202.5 | 205.2 | **203.7** | 1.4 | 0.7 | 4.2 |
| INE963 M2 | Hydroxylation + demethylation | [M+H]^+^ | 405.1658 | **195.5** | 201.3 | --- | --- | **201.3** | --- | --- | 3.0 |
| INE963 M7 | Demethylation | [M+H]^+^ | 389.1699 | **195.9** | 197.3 | --- | --- | **197.3** | --- | --- | 0.7 |
| INE963 M11 | O-glucuronidation | [M+H]^+^ | 579.2252 | **229.6** | 246.3 | --- | --- | **246.3** | --- | --- | 7.3 |
| INE963 M13 | Hydroxylation | [M+H]^+^ | 419.1809 | **197.2** | 207.3 | --- | --- | **207.3** | --- | --- | 5.1 |
| INE963 M16 | Hydroxylation + N-acetylation | [M+H]^+^ | 461.1926 | **207.8** | 220.8 | --- | --- | **220.8** | --- | --- | 6.3 |
| Lansoprazole | Parent drug | [M+H]^+^ | 370.0828 | **177.2** | 179.2 | 179.3 | 179.0 | **179.2** | 0.2 | 0.1 | 1.1 |
| Lansoprazole N-oxide | N-oxidation | [M+H]^+^ | 386.0790 | **183.8** | 179.7 | 180.2 | 180.2 | **180.0** | 0.3 | 0.2 | -2.0 |
| Lansoprazole sulfide | Sulfoxide reduction | [M+H]^+^ | 354.0897 | **176.7** | 176.2 | 176.8 | 176.6 | **176.5** | 0.3 | 0.2 | -0.1 |
| Lansoprazole sulfone | Sulfoxide oxygenation | [M+H]^+^ | 386.0789 | **183.1** | 182.8 | 182.7 | 183.2 | **182.9** | 0.3 | 0.1 | -0.1 |
| Lansoprazole sulfone N-oxide | Sulfoxide oxygenation + N-oxidation | [M+H]^+^ | 402.0740 | **186.1** | 183.6 | 183.3 | 183.8 | **183.6** | 0.2 | 0.1 | -1.4 |
| Losartan | Parent drug | [M+H]^+^ | 423.1702 | **199.9** | 198.1 | 198.2 | 198.3 | **198.2** | 0.1 | 0.1 | -0.9 |
| Losartan N-2-glucuronide | N-glucuronidation | [M+H]^+^ | 599.2023 | **235.2** | 232.4 | 232.3 | 235.4 | **233.4** | 1.8 | 0.8 | -0.8 |
| MAK683 | Parent drug | [M+H]^+^ | 377.1521 | **189.2** | 196.4 | 196.4 | 196.3 | **196.4** | <0.1 | <0.1 | 3.8 |
| ^a^ Waters CCSonDemand, ^b^ SD: standard deviation, ^c^ CV: coefficient of variation, ^d^ Bias (%) = 100 x (mean ^TW^CCS_N2, meas_ - ^TW^CCS_N2, pred_) / ^TW^CCS_N2, pred_, ^e^ generated following *in vitro* incubation of parent drug / precursor | | | | | | | | | | | |

**Table S5** continued

| **Compound** | **Biotransformation** | **Ion** | ***m/z*** | **^TW^CCS_N2, pred_**  **(Å^2^)^a^** | **^TW^CCS_N2, meas_ (Å^2^)** | | | | | | **Bias**  **(%)^d^** |
| --- | --- | --- | --- | --- | --- | --- | --- | --- | --- | --- | --- |
|  |  |  |  |  | 1 | 2 | 3 | Mean | SD^b^ | CV^c^ |  |
| MAK683 M26 | Ring opening + carboxylic acid formation | [M+H]^+^ | 409.1419 | **196.0** | 199.0 | 198.9 | 198.9 | **198.9** | 0.1 | <0.1 | 1.5 |
| MAK683 M47 | N-oxidation + hydroxylation | [M+H]^+^ | 409.1419 | **196.4** | 200.5 | 200.5 | 200.5 | **200.5** | <0.1 | <0.1 | 2.1 |
| MAK683 M48 | Hydroxylation + N-oxidation | [M+H]^+^ | 409.1419 | **196.5** | 199.9 | 199.8 | --- | **199.8** | 0.1 | <0.1 | 1.7 |
| Mavoglurant | Parent drug | [M+Na]^+^ | 336.1566 | **183.6** | 185.2 | 185.2 | 184.6 | **185.0** | 0.3 | 0.2 | 0.8 |
| Mavoglurant M5 | O-glucuronidation | [M+Na]^+^ | 512.1755 | **213.8** | 218.9 | 218.8 | 218.7 | **218.8** | 0.1 | <0.1 | 2.3 |
| Mephenytoin | Parent drug | [M+H]^+^ | 219.1140 | **146.7** | 146.9 | 146.8 | 146.9 | **146.9** | 0.1 | <0.1 | 0.1 |
| Midazolam | Parent drug | [M+H]^+^ | 326.0864 | **173.9** | 167.0 | 167.6 | 167.0 | **167.2** | 0.4 | 0.2 | -3.9 |
| N-acetyl sulfamonomethoxine | Acetylation | [M+H]^+^ | 323.0806 | **171.1** | 175.5 | 175.6 | 175.6 | **175.5** | 0.1 | <0.1 | 2.6 |
| N-acetyl trovafloxacin | Acetylation | [M+H]^+^ | 459.1270 | **202.8** | 203.4 | 203.0 | 202.9 | **203.1** | 0.3 | 0.1 | 0.1 |
| Naloxone | Parent drug | [M+H]^+^ | 328.1552 | **170.9** | 170.1 | 170.3 | 170.1 | **170.2** | 0.1 | 0.1 | -0.4 |
| Naloxone-3-glucuronide | O-glucuronidation | [M+H]^+^ | 504.1869 | **211.0** | 217.7 | 218.2 | 218.7 | **218.2** | 0.5 | 0.2 | 3.4 |
| N-desmethyl tamoxifen | Demethylation | [M+H]^+^ | 358.2169 | **196.8** | 183.7 | 184.3 | 184.2 | **184.1** | 0.3 | 0.2 | -6.5 |
| Nordazepam | Demethylation | [M+H]^+^ | 271.0642 | **159.9** | 153.1 | 153.4 | 152.9 | **153.2** | 0.3 | 0.2 | -4.2 |
| Nortestosterone | Demethylation | [M+H]^+^ | 275.2013 | **168.4** | 164.5 | 165.1 | 165.1 | **164.9** | 0.3 | 0.2 | -2.1 |
| NVS1 | Parent drug | [M+H]^+^ | --- | **198.2** | 208.6 | 208.3 | 208.2 | **208.4** | 0.2 | 0.1 | 5.1 |
| NVS1 M1 | Hydroxylation | [M+H]^+^ | --- | **200.5** | 216.3 | 213.9 | 213.7 | **214.6** | 1.4 | 0.7 | 7.0 |
| NVS1 M2 | O-glucuronidation | [M+H]^+^ | --- | **224.2** | 233.8 | 234.2 | 234.7 | **234.2** | 0.5 | 0.2 | 4.5 |
| NVS1 M3 | N-glucuronidation | [M+H]^+^ | --- | **228.7** | 250.2 | 250.5 | 249.1 | **249.9** | 0.7 | 0.3 | 9.3 |
| NVS1 M4 | N-glucuronidation | [M+H]^+^ | --- | **228.6** | 250.4 | 250.2 | 251.8 | **250.8** | 0.9 | 0.3 | 9.7 |
| NVS1 M5 | N-glucuronidation | [M+H]^+^ | --- | **222.2** | 249.9 | 248.2 | 247.9 | **248.7** | 1.1 | 0.4 | 11.9 |
| NVS1 M6 | Sulfation | [M+H]^+^ | --- | **212.3** | 219.3 | 218.3 | 218.3 | **218.6** | 0.6 | 0.3 | 3.0 |
| NVS1 M7 | Acetylation | [M+H]^+^ | --- | **208.7** | 220.4 | 220.5 | 217.5 | **219.5** | 1.7 | 0.8 | 5.2 |
| NVS1 M8 | Ring opening + carboxylic acid formation | [M+H]^+^ | --- | **203.8** | 212.1 | 212.6 | 212.7 | **212.4** | 0.3 | 0.1 | 4.2 |
| NVS2 | Parent drug | [M-H]^-^ | --- | **185.7** | 175.8 | 177.2 | 175.6 | **176.2** | 0.9 | 0.5 | -5.1 |
| NVS2 M1 | Sulfation | [M-H]^-^ | --- | **197.0** | 188.0 | 188.2 | 188.3 | **188.1** | 0.2 | 0.1 | -4.5 |
| NVS3 | Parent drug | [M+H]^+^ | --- | **206.5** | 196.7 | 196.7 | 196.7 | **196.7** | <0.1 | <0.1 | -4.8 |
| NVS3 M1 | Hydroxylation | [M+H]^+^ | --- | **205.3** | 200.4 | 200.2 | 202.8 | **201.1** | 1.5 | 0.7 | -2.0 |
| NVS4 | Parent drug | [M+H]^+^ | --- | **243.1** | 244.3 | 244.6 | --- | **244.5** | 0.2 | 0.1 | 0.6 |
| NVS4 M1 | Hydroxylation | [M+H]^+^ | --- | **243.7** | 248.3 | 248.7 | --- | **248.5** | 0.3 | 0.1 | 2.0 |
| NVS4 M2 | Didemethylation + dihydroxylation | [M+H]^+^ | --- | **244.4** | 249.9 | 250.3 | --- | **250.1** | 0.2 | 0.1 | 2.3 |
| NVS5 | Parent drug | [M+H]^+^ | --- | **217.3** | 206.3 | 206.1 | 206.3 | **206.2** | 0.2 | 0.1 | -5.1 |
| NVS5 M1 | Demethylation | [M+H]^+^ | --- | **204.9** | 199.9 | 200.0 | 200.1 | **200.0** | 0.1 | <0.1 | -2.4 |
| NVS5 M2 | Demethylation | [M+H]^+^ | --- | **206.5** | 200.7 | 200.7 | 200.7 | **200.7** | <0.1 | <0.1 | -2.8 |
| NVS5 M3 | Amide hydrolysis | [M+H]^+^ | --- | **167.5** | 173.5 | 173.6 | 173.7 | **173.6** | 0.1 | <0.1 | 3.6 |
| NVS5 M4 | Demethylation + amide hydrolysis | [M+H]^+^ | --- | **204.9** | 204.6 | 204.6 | 204.6 | **204.6** | <0.1 | <0.1 | -0.1 |
| NVS5 M5 | Amide hydrolysis | [M+H]^+^ | --- | **138.1** | 147.0 | 147.0 | 147.0 | **147.0** | <0.1 | <0.1 | 6.4 |
| NVS6 | Parent drug | [M+H]^+^ | --- | **215.4** | 223.1 | 223.2 | 223.2 | **223.2** | 0.0 | <0.1 | 3.6 |
| NVS6 M1 | Dealkylation | [M+H]^+^ | --- | **195.7** | 196.5 | 196.5 | 196.4 | **196.5** | 0.1 | <0.1 | 0.4 |
| NVS6 M2 | Dihydrodiol formation | [M+H]^+^ | --- | **230.8** | 229.8 | 229.5 | 230.1 | **229.8** | 0.3 | 0.1 | -0.4 |
| NVS7 | Parent drug | [M+H]^+^ | --- | **204.9** | 214.1 | 214.1 | 214.3 | **214.2** | 0.1 | <0.1 | 4.5 |
| NVS8 | Hydroxylation | [M+H]^+^ | --- | **244.0** | 244.2 | 244.1 | 244.2 | **244.1** | 0.1 | <0.1 | 0.1 |
| NVS9 | Dihydroxylation | [M+H]^+^ | --- | **242.5** | 243.8 | 243.9 | 243.8 | **243.8** | <0.1 | <0.1 | 0.5 |
| ^a^ Waters CCSonDemand, ^b^ SD: standard deviation, ^c^ CV: coefficient of variation, ^d^ Bias (%) = 100 x (mean ^TW^CCS_N2, meas_ - ^TW^CCS_N2, pred_) / ^TW^CCS_N2, pred_, ^e^ generated following *in vitro* incubation of parent drug / precursor | | | | | | | | | | | |

**Table S5** continued

| **Compound** | **Biotransformation** | **Ion** | ***m/z*** | **^TW^CCS_N2, pred_**  **(Å^2^)^a^** | **^TW^CCS_N2, meas_ (Å^2^)** | | | | | | **Bias**  **(%)^d^** |
| --- | --- | --- | --- | --- | --- | --- | --- | --- | --- | --- | --- |
|  |  |  |  |  | 1 | 2 | 3 | Mean | SD^b^ | CV^c^ |  |
| NVS10 | Hydroxylation | [M+H]^+^ | --- | **246.7** | 251.1 | 251.1 | 251.2 | **251.2** | 0.1 | <0.1 | 1.8 |
| NVS11 | Hydroxylation | [M+H]^+^ | --- | **204.8** | 194.1 | 193.8 | --- | **193.9** | 0.2 | 0.1 | -5.3 |
| Olodanrigan | Parent drug | [M-H]^-^ | 506.1973 | **224.8** | 226.9 | 226.6 | 227.0 | **226.8** | 0.2 | 0.1 | 0.9 |
| Olodanrigan M10 | O-debenzylation + glucuronidation | [M-H]^-^ | 592.1823 | **233.6** | 222.3 | 221.8 | 223.1 | **222.4** | 0.7 | 0.3 | -4.8 |
| Omeprazole | Parent drug | [M+H]^+^ | 346.1240 | **180.2** | 178.7 | 178.7 | 179.0 | **178.8** | 0.2 | 0.1 | -0.8 |
| Pantoprazole | Parent drug | [M+H]^+^ | 384.0832 | **182.2** | 183.1 | 183.1 | 182.8 | **183.0** | 0.2 | 0.1 | 0.4 |
| Pantoprazole N-oxide | N-oxidation | [M+H]^+^ | 400.0783 | **186.9** | 182.9 | 182.9 | 183.0 | **182.9** | 0.1 | <0.1 | -2.1 |
| Raloxifene | Parent drug | [M+H]^+^ | 474.1736 | **224.1** | 213.9 | 213.9 | 214.1 | **214.0** | 0.1 | 0.1 | -4.5 |
| Raloxifene 4'-glucuronide | O-glucuronidation | [M+H]^+^ | 650.2060 | **250.0** | 250.8 | 251.0 | 248.7 | **250.2** | 1.3 | 0.5 | 0.1 |
| Raloxifene sulfate^e^ | Sulfation | [M+H]^+^ | 554.1299 | **227.7** | 232.8 | 233.7 | 232.8 | **233.1** | 0.5 | 0.2 | 2.4 |
| Ranitidine | Parent drug | [M+H]^+^ | 315.1491 | **169.2** | 167.2 | 166.7 | 166.6 | **166.8** | 0.3 | 0.2 | -1.4 |
| Ranitidine N-oxide | N-oxidation | [M+H]^+^ | 331.1429 | **172.2** | 169.9 | 169.9 | 170.0 | **169.9** | 0.1 | <0.1 | -1.3 |
| Ranitinide S-oxide | Oxygenation | [M+H]^+^ | 331.1437 | **175.2** | 167.1 | 167.7 | 167.1 | **167.3** | 0.4 | 0.2 | -4.5 |
| Ranitinide N, S-dioxide | Oxygenation + N-oxidation | [M+H]^+^ | 347.1380 | **176.4** | 170.7 | 171.2 | 170.4 | **170.8** | 0.4 | 0.2 | -3.2 |
| Repaglinide | Parent drug | [M+H]^+^ | 453.2754 | **219.5** | 213.6 | 212.6 | 212.9 | **213.0** | 0.5 | 0.2 | -2.9 |
| Repaglinide N-oxide | N-oxidation | [M+H]^+^ | 469.2695 | **219.0** | 216.8 | 216.7 | 216.8 | **216.8** | 0.1 | <0.1 | -1.0 |
| Resveratrol | Parent drug | [M+H]^+^ | 229.0866 | **152.8** | 151.3 | 151.2 | 151.0 | **151.2** | 0.2 | 0.1 | -1.1 |
| Resveratrol O-sulfate | Sulfation | [M+H]^+^ | 309.0434 | **168.4** | 171.7 | 171.6 | 171.2 | **171.5** | 0.3 | 0.2 | 1.8 |
| Ribociclib | Parent drug | [M+H]^+^ | 435.2613 | **214.6** | 219.9 | 220.0 | 219.7 | **219.9** | 0.1 | 0.1 | 2.4 |
| Ribociclib M8 | Sulfation | [M+H]^+^ | 515.2189 | **221.3** | 243.3 | 243.7 | 243.1 | **243.3** | 0.3 | 0.1 | 10.0 |
| Rociletinib | Parent drug | [M+H]^+^ | 556.2274 | **232.4** | 226.8 | 226.7 | 226.9 | **226.8** | 0.1 | <0.1 | -2.4 |
| Rociletinib glutathione^e^ | Glutathione conjugation | [M+H]^+^ | 863.3115 | **269.6** | 272.0 | 272.0 | 272.1 | **272.0** | 0.1 | <0.1 | 0.9 |
| Salbutamol | Parent drug | [M+H]^+^ | 240.1604 | **159.3** | 157.2 | 157.2 | 156.8 | **157.1** | 0.2 | 0.1 | -1.4 |
| Serotonin | Parent drug | [M-H]^-^ | 175.0868 | **136.8** | 135.6 | 135.5 | 135.6 | **135.6** | 0.1 | <0.1 | -0.9 |
| Serotonin sulfate^e^ | Sulfation | [M-H]^-^ | 255.0790 | **151.9** | 155.0 | 154.7 | 154.7 | **154.8** | 0.2 | 0.1 | 1.9 |
| Sotuletinib | Parent drug | [M+H]^+^ | 399.1492 | **195.4** | 205.9 | 207.0 | 205.8 | **206.2** | 0.6 | 0.3 | 5.5 |
| Sotuletinib M6 | O-glucuronidation | [M+H]^+^ | 575.1815 | **234.5** | 230.4 | 230.7 | 229.4 | **230.2** | 0.7 | 0.3 | -1.8 |
| Spebrutinib | Parent drug | [M+H]^+^ | 424.1777 | **195.6** | 207.1 | 207.1 | 207.6 | **207.3** | 0.3 | 0.1 | 6.0 |
| Spebrutinib glutathione^e^ | Glutathione conjugation | [M+H]^+^ | 731.2607 | **252.8** | 249.8 | 249.4 | 249.4 | **249.5** | 0.2 | 0.1 | -1.3 |
| Sulfamonomethoxine | Parent drug | [M+H]^+^ | 281.0709 | **160.3** | 160.4 | 160.3 | 160.2 | **160.3** | 0.1 | 0.1 | <0.1 |
| Tamoxifen | Parent drug | [M+H]^+^ | 372.2325 | **200.2** | 195.3 | 195.8 | 196.2 | **195.8** | 0.5 | 0.2 | -2.2 |
| Tamoxifen N-glucuronide | N-glucuronidation | [M+H]^+^ | 548.2647 | **236.5** | 231.1 | 232.0 | 232.4 | **231.8** | 0.7 | 0.3 | -2.0 |
| Tamoxifen N-oxide | N-oxidation | [M+H]^+^ | 388.2273 | **203.3** | 205.6 | 206.2 | 207.3 | **206.4** | 0.8 | 0.4 | 1.5 |
| Testosterone | Parent drug | [M+H]^+^ | 289.2170 | **172.6** | 168.3 | 168.8 | 168.1 | **168.4** | 0.4 | 0.2 | -2.4 |
| Testosterone β-D-glucuronide | O-glucuronidation | [M+H]^+^ | 465.2489 | **208.8** | 217.4 | 218.4 | 217.4 | **217.7** | 0.6 | 0.3 | 4.3 |
| Triclosan | Parent drug | [M-H]^-^ | 286.9446 | **157.0** | 152.6 | 152.5 | 152.2 | **152.4** | 0.2 | 0.1 | -2.9 |
| Triclosan O-sulfate | Sulfation | [M-H]^-^ | 366.9015 | **169.9** | 165.7 | 165.9 | 165.6 | **165.7** | 0.2 | 0.1 | -2.5 |
| Triclosan O-β-D-glucuronide | O-glucuronidation | [M-H]^-^ | 462.9779 | **200.7** | 191.9 | 190.2 | 191.5 | **191.2** | 0.9 | 0.5 | -4.7 |
| Trovafloxacin | Parent drug | [M+H]^+^ | 417.1166 | **198.3** | 191.0 | 191.0 | 191.0 | **191.0** | <0.1 | <0.1 | -3.7 |
| Verapamil | Parent drug | [M+H]^+^ | 455.2910 | **215.5** | 208.7 | 208.7 | 209.0 | **208.8** | 0.2 | 0.1 | -3.1 |
| ^a^ Waters CCSonDemand, ^b^ SD: standard deviation, ^c^ CV: coefficient of variation, ^d^ Bias (%) = 100 x (mean ^TW^CCS_N2, meas_ - ^TW^CCS_N2, pred_) / ^TW^CCS_N2, pred_, ^e^ generated following *in vitro* incubation of parent drug / precursor | | | | | | | | | | | |

**Table S6** Summary of investigated compound pairs (n=86) covering eight common types of phase I and II biotransformation

| **Hydroxylation (n=19)** | |  | **N-oxidation (n=9)** | |  | **Demethylation (n=13)** | |  | **O-glucuronidation (n=14)** | |
| --- | --- | --- | --- | --- | --- | --- | --- | --- | --- | --- |
|  |  |  |  |  |  |  |  |  |  |  |
| **Parent / precursor** | **Metabolite** |  | **Parent / precursor** | **Metabolite** |  | **Parent / precursor** | **Metabolite** |  | **Parent / precursor** | **Metabolite** |
| Bupropion | Hydroxy bupropion |  | Amitriptyline | Amitriptyline N-oxide |  | 4-hydroxy tamoxifen | Endoxifen |  | 4-hydroxy propanolol | 4-hydroxy propanolol glucuronide |
| Chlorzoxazone | 6-hydroxy chlorzoxazone |  | Clozapine | Clozapine N-oxide |  | Clozapine | Desmethyl clozapine |  | 7-hydroxy coumarin | 7-hydroxy coumarin glucuronide |
| Coumarin | 7-hydroxy coumarin |  | Imipramine | Imipramine N-oxide |  | Dextromethorphan | Dextrorphan |  | Acetaminophen | 4-acetamidophenyl β-D-glucuronide |
| Diclofenac | 4-hydroxy diclofenac |  | Lansoprazole | Lansoprazole N-oxide |  | Diazepam | Nordazepam |  | EMA1323 | Olodanrigan M10 |
| Diclofenac | 5-hydroxy diclofenac |  | Lansoprazole sulfone | Lansoprazole sulfone N-oxide |  | EMA1323 | EMA1322 |  | Estradiol | Estradiol-3-glucuronide |
| Estradiol | 4-hydroxy estradiol |  | Pantoprazole | Pantoprazole N-oxide |  | Granisetron | 9'-desmethyl granisetron |  | Ezetimibe | Ezetimibe phenoxy-β-glucuronide |
| Ibuprofen | 1-hydroxy ibuprofen |  | Ranitidine | Ranitidine N-oxide |  | INE963 | INE963 M7 |  | INE963 | INE963 M11 |
| Imipramine | 2-hydroxy imipramine |  | Repaglinide | Repaglinide N-oxide |  | INE963 M13 | INE963 M2 |  | Mavoglurant | Mavoglurant M5 |
| Imipramine | 10-hydroxy imipramine |  | Tamoxifen | Tamoxifen N-oxide |  | NVS5 | NVS5 M1 |  | Naloxone | Naloxone-3-glucuronide |
| INE963 | INE963 M13 |  |  |  |  | NVS5 | NVS5 M2 |  | NVS1 | NVS1 M2 |
| Lansoprazole | 5-hydroxy lansoprazole |  |  |  |  | Olodanrigan | EMA1036 |  | Raloxifene | Raloxifene 4'-glucuronide |
| Mephenytoin | 4-hydroxy mephenytoin |  |  |  |  | Tamoxifen | N-desmethyl tamoxifen |  | Sotuletinib | Sotuletinib M6 |
| Midazolam | 1-hydroxy midazolam |  |  |  |  | Testosterone | Nortestosterone |  | Testosterone | Testosterone β-D-glucuronide |
| NVS1 | NVS1 M1 |  |  |  |  |  |  |  | Triclosan | Triclosan O-β-D-glucuronide |
| NVS3 | NVS3 M1 |  |  |  |  |  |  |  |  |  |
| NVS4 | NVS4 M1 |  |  |  |  |  |  |  |  |  |
| Omeprazole | 5-hydroxy omeprazole |  |  |  |  |  |  |  |  |  |
| Tamoxifen | 4-hydroxy tamoxifen |  |  |  |  |  |  |  |  |  |
| Testosterone | 6-β-hydroxy testosterone |  |  |  |  |  |  |  |  |  |
|  |  |  |  |  |  |  |  |  |  |  |
|  |  |  |  |  |  |  |  |  |  |  |
| **N-glucuronidation (n=9)** | |  | **Sulfation (n=10)** | |  | **Glutathione conjugation (n=7)** | |  | **Acetylation (n=5)** | |
|  |  |  |  |  |  |  |  |  |  |  |
| **Parent / precursor** | **Metabolite** |  | **Parent / precursor** | **Metabolite** |  | **Parent / precursor** | **Metabolite** |  | **Parent / precursor** | **Metabolite** |
| Amitriptyline | Amitriptyline N-glucuronide |  | 7-hydroxy coumarin | 7-hydroxy coumarin sulfate^a^ |  | Acetaminophen | Acetaminophen glutathione |  | INE963 M13 | INE963 M16 |
| Chlorzoxazone | Chlorzoxazone N-glucuronide |  | Acetaminophen | 4-acetaminophen sulfate |  | Acalabrutinib | Acalabrutinib glutathione^a^ |  | Infigratinib M1 (CQM157) | Infigratinib M35 |
| Dapsone | Dapsone N-glucuronide |  | Estradiol | Estradiol-3-sulfate^a^ |  | Afatinib | Afatinib glutathione^a^ |  | NVS1 | NVS1 M7 |
| Icenticaftor | Icenticaftor M1 |  | NVS1 | NVS1 M6 |  | Branebrutinib | Branebrutinib glutathione^a^ |  | Sulfamonomethoxine | N-acetyl sulfamonomethoxine |
| Losartan | Losartan N-2-glucuronide |  | NVS2 | NVS2 M1 |  | Ibrutinib | Ibrutinib glutathione^a^ |  | Trovafloxacin | N-acetyl trovafloxacin |
| NVS1 | NVS1 M3 |  | Raloxifene | Raloxifene sulfate^a^ |  | Rociletinib | Rociletinib glutathione^a^ |  |  |  |
| NVS1 | NVS1 M4 |  | Resveratrol | Resveratrol O-sulfate |  | Spebrutinib | Spebrutinib glutathione^a^ |  |  |  |
| NVS1 | NVS1 M5 |  | Ribociclib | Ribociclib M8 |  |  | |  |  |  |
| Tamoxifen | Tamoxifen N-glucuronide |  | Serotonin | Serotonin sulfate^a^ |  |  |  |  |  |  |
|  |  |  | Triclosan | Triclosan O-sulfate |  |  |  |  |  |  |
|  | | | | |  |  |  |  |  |  |
| ^a^ Following *in vitro* incubation of parent drug / precursor | | | | |  |  |  |  |  |  |

**Fig. S2** Comparison of relative mean shifts in ^TW^CCS_N2_ based on predicted and measured values

**
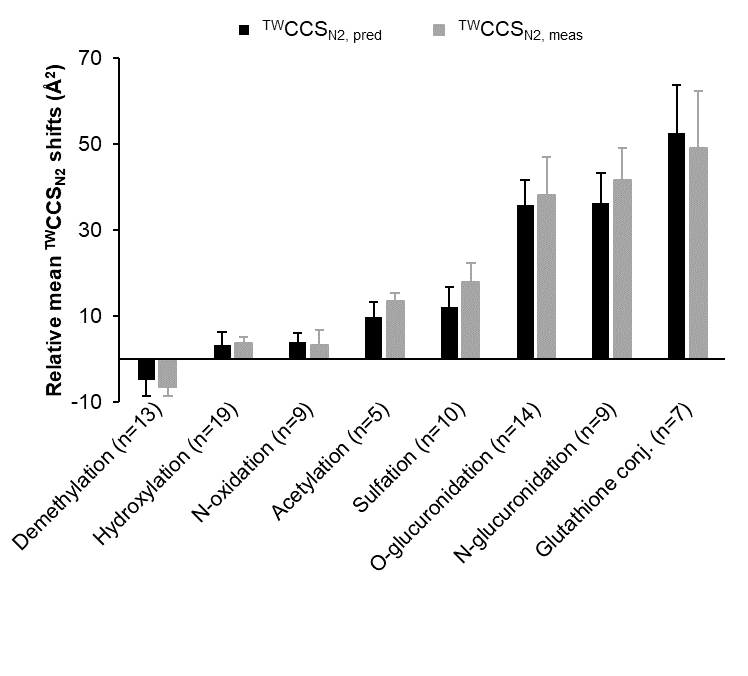
**
